# Zn_3_(PO_4_)_2_ shell effects on Zn uptake and cellular distribution of root applied ZnO NPs†

**DOI:** 10.1039/d5en00217f

**Published:** 2025-05-29

**Authors:** Sandra Rodrigues, Astrid Avellan, Hiram Castillo-Michel, Matheus C. R. Miranda, Diana Salvador, Aude Calas, Gregory V. Lowry, Sónia M. Rodrigues

**Affiliations:** a Centre for Environmental and Marine Studies (CESAM), Department of Environment and Planning, Universidade de Aveiro 3810-193 Aveiro Portugal rodriguesandra.research@gmail.com; b Géosciences-Environnement-Toulouse (GET), CNRS, UMR 5563 CNRS, UT3, IRD, CNES, OMP 31400 Toulouse France; c ESRF, The European Synchrotron 71 Avenue des Martyrs, CS40220 38043 Grenoble Cedex 9 France; d Centre for Environmental and Marine Studies (CESAM), Department of Chemistry, Universidade de Aveiro 3810-193 Aveiro Portugal; e Centre for Environmental and Marine Studies (CESAM), Department of Biology, Universidade de Aveiro 3810-193 Aveiro Portugal; f Department of Civil and Environmental Engineering, Carnegie Mellon University Pittsburgh Pennsylvania 15213 USA

## Abstract

Touted benefits of nanoparticle-based fertilizers include enhancing crop nutrition by fortifying fruits or grains with nutrient metals and reducing environmental impacts of fertilizer use. However, the properties of the nanoparticles (NPs) and application routes required to achieve these benefits are not yet established. This study examined how a Zn-phosphate shell on ZnO NPs (ZnO_Ph NPs) affected root uptake, cellular distribution, transformation, and translocation of Zn in pepper plants (*Capsicum annuum*), and compared the efficacy of root- to foliarly-applied NPs. Pepper plants roots were exposed to ZnO NPs (26 ± 8 nm), ZnO_Ph NPs (48 ± 12 nm), or ionic Zn. After 6 weeks, 30–37% of root-applied Zn was absorbed, with 6.0–7.2% (2.4–2.9 μg) reaching the fruits. ZnO_Ph NPs resulted in lower total Zn uptake, but higher mobility into the root vasculature and stem epidermis, likely due to P–Zn co-delivery modulating translocation mechanisms. Foliar application of these NPs led to lower Zn uptake (2.4% for ZnO_Ph NPs; 0.5% for ZnO NPs) compared to root application. However, a greater proportion of the Zn that was taken up for foliar-applied ZnO_Ph NP translocated to the fruits (27%) compared to root application (10%). Root and foliar applications also led to contrasting Zn speciation in the stem vasculature. Foliar-applied Zn formed only carboxyl and phytate-like complexes, while root-applied Zn also formed Zn–S–R complexes, indicating distinct Zn transport and storage responses, possibly explaining the higher relative mobility to the fruits when foliar-applied. These findings demonstrate that Zn uptake efficiency and speciation depend on both application method and nanoparticle formulation. They also suggest that multi-nutrient NPs can fortify foods, potentially offering a new strategy for improving plant nutrition.

## Abstract 2

ZnO naoparticles (NPs) with a Zn-phosphate shell can modulate the routes of Zn root uptake, translocation and storage mechanisms compared to ZnO NPs. Applying ZnO NPs to roots provides much greater uptake into plants than for foliar application.

## Graphical abstract

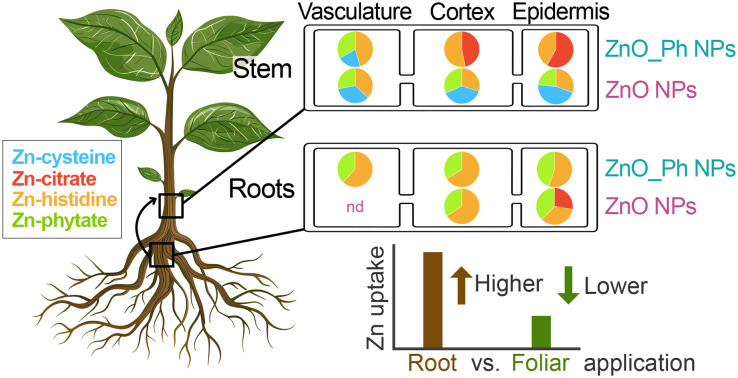

**Environmental significance**

These results show that applying ZnO NPs to roots provides greater uptake into plants than for foliar applications, which has broad implications for promoting efficient use of micronutrient fertilizers. It also shows that designing NPs with multiple plant nutrients (Zn and P together) can modulate foliar routes of uptake and Zn translocation and storage mechanisms compared to Zn alone, potentially enabling more efficient NP designs for targeting specific plant organs or fruits.

## 1. Introduction

Soil zinc (Zn) deficiency is a common issue that affects agricultural lands worldwide, which consequently affects food nutritional value^1^ and can impair human health.^2^ As a result, Zn fertilizers are often added to soils as soluble or chelated forms (ZnSO_4_, Zn-EDTA or ZnCl_2_),^1,3^ but depending on soil characteristics, only a small fraction of Zn applied to soil is bioavailable for root uptake.^4,5^ Soil pH highly influences Zn bioavailability for plants. Acidic soils (pH < 5.5) increase Zn^2+^ solubility and bioavailability, but this also increases Zn losses though leaching and downward migration.^6,7^ However, for soils with pH 5.5–7.0, Zn bioavailability decreases by 30 to 45-fold, compared to more acidic soils^6^ due to the formation of low solubility precipitates with phosphates, carbonates or hydroxides^8^ in soil, rendering Zn unavailable for plant root uptake.^1^ These limitations pose a challenge for Zn bioavailability and plant uptake. Nano-enabled strategies are an alternative to conventional fertilizers which could enhance nutrient delivery to plants, thus increasing fertilization efficiency and possibly leading to higher crop yields.^9^ Enabling a more targeted delivery system to plants would reduce the amount of resources used for fertilization, while minimizing environmental impacts.^10,11^

ZnO nanoparticle (NP)-based fertilizers are being developed to improve Zn^2+^ root or foliar uptake in plants while also minimizing losses of soluble Zn^2+^ to the soil. The approach in acidic soils is to attach ZnO NP to plant roots where a slow rate of dissolution provides plants with Zn^2+^ at the root surface at a rate that minimizes Zn^2+^ losses in acidic soils. In basic soils where Zn is insoluble and not bioavailable, the approach is to use surface functionalization to enable ZnO NP uptake into roots where it can dissolve and provide Zn to the plants.^12–15^ Root uptake can also be improved using inorganic coatings that both prevent NP dissolution and promote Zn root association, uptake and translocation in plants.^16–18^ However, in some cases strong NP association to plant roots (*e.g.* ZnO NPs^19^) has been shown to limit immediate NP uptake.^20,21^ Foliar application of ZnO NPs with a Zn-phosphate shell enhanced Zn foliar uptake, phloem loading, and translocation in pepper plants compared to bare ZnO NPs.^18^ The effects of adding a Zn-phosphate shell on ZnO NPs when root applied and unravelling the associated routes of uptake and translocation have not been explored. Further, quantitatively assessing the efficiency of uptake of ZnO NPs between foliar and root applied NPs is yet to be performed, although this type of comparison is needed to develop more efficient nano-based fertilizers.

Better understanding how Zn speciation of Zn-based NPs affects Zn root uptake is needed to design efficient fertilizers. Phosphate plays a role in Zn solubility in soil, which consequently affects its bioavailability for plant uptake and *in planta* translocation.^22–24^ The presence of orthophosphates in soils at alkaline pH inhibits Zn bioavailability by forming poorly soluble Zn-phosphate precipitates,^5,25–27^ while under acidic conditions (pH < 7), Zn-phosphate transforms to more soluble forms of Zn such as Zn dihydrogen phosphate (Zn(H_2_PO_4_)_2_).^28^ Lv *et al.*^29^ showed that Zn^2+^ release from ZnO NPs dissolution was drastically diminished by adding phosphate in the suspension medium, through the formation of Zn-phosphate precipitates on the particle surfaces. Using a Zn-phosphate shell on ZnO NPs could therefore potentially play a role in improving Zn root uptake by either lowering ZnO NPs dissolution and/or triggering Zn uptake along with phosphate uptake.^17^ Differences in Zn complexation that results from the dissolution of applied Zn-based nano-formulations at the root surface (Zn^2+^_(aq)_ and other different Zn forms *e.g.* Zn(OH)_2(aq)_; Zn_3_(PO_4_)^2−^_(aq)_)^30^ could hypothetically be created to provide the desired Zn association to roots and desired Zn uptake pathway.^31^

Delivering phosphate along with Zn could also influence Zn translocation in plants, and thus its capacity to reach the target plant organs.^26,27,32–34^ Plants grown under Zn deficient conditions enhanced inorganic phosphate (Pi) root uptake and translocation to the shoots due an impaired control of Pi-gene activation of protein transporters towards the xylem and Pi remobilization in the phloem.^27,33^ The described connection between Zn and Pi uptake implies that NPs that would provide both nutrients simultaneously may engage different translocation and storage pathways compared to those that deliver Zn without P. Our previous work^18^ using foliarly applied ZnO-based NPs in pepper plants grown in a Zn deficient medium showed that ZnO NPs covered with an amorphous and heterogeneous Zn_3_(PO_4_)_2_ shell had higher Zn translocation *in planta* than uncoated ZnO NPs. This study suggested that the presence of Zn_3_(PO_4_)_2_ precipitates on the ZnO NPs when applied to the surface of the leaves affected Zn uptake, cellular distribution, and the plant's Zn storage strategy.^18^ However, it remains unclear if and how formulating these two elements in a nanomaterial delivered to roots could improve Zn biofortification in crops.

Foliar and root application are both proposed as application routes for NP nutrient formulations. Zn absorption through the roots occurs by crossing the root epidermis and Casparian strip, while in foliar application, NPs must cross several barriers before being taken up, which can affect Zn fertilizer effectiveness (*e.g.* cuticle, epidermis, stomata).^35,36^ Comparison of the uptake and translocation efficacy combined with in-depth biotransformation differences in plant tissues between root *vs.* foliar exposure of the same NPs on the same plant species at the same application rates is scarce.^37^ In existent comparative studies significantly higher amounts for soil application when compared to the foliar application are often used,^38^ which can muddle conclusions in terms of comparing uptake and translocation efficiencies. Furthermore, comparing the μ-XRF imaging of Zn uptake and translocation to other tissues (or the product of NP dissolution) to biotransformation analysis, provides a more complete understanding of the possible pathways involved between application modes. Comparative studies between foliar and root application of ZnO-based NPs are needed to quantify the differences in the cellular processes involved in uptake and translocation. This understanding is needed to determine the most efficient route of application and to optimize the NP physico-chemical properties that enable targeted Zn delivery.

To address these knowledge gaps, this study aims to (1) explore the mechanisms that control the uptake, cellular distribution, and translocation of Zn in root and stem tissues, (2) determine differences in Zn delivery between ZnO NP and ZnO NPs with an amorphous Zn_3_(PO_4_)_2_ shell, and (3) quantify the differences in the efficacy of root applied *vs.* foliar applied NPs in pepper plants. Zn uptake, translocation and biotransformation was assessed after exposing pepper plants roots to ZnO NPs with and without a Zn_3_(PO_4_)_2_ shell in a Zn deficient medium. The resulting Zn uptake, translocation, and speciation in plant tissues was determined. Results for root application of the Zn-based NPs was compared with that previously determined for foliar application of these particles^18^ to quantify the benefits and limitations of these two application methods. The present study will determine which Zn application strategy (foliar *vs.* root) is more efficient and better able to fortify the fruits by delivering Zn into the edible plant parts. It will determine if changing the surface chemistry of ZnO NPs with a Zn-phosphate shell and the dissolution behavior of these NPs presents an opportunity for improving the efficacy of Zn-based NP delivery.

## 2. Materials and methods

### 2.1. ^68^ZnO-based NP syntheses and characterization

Two ZnO-based NPs were used in this study: 1) bare ZnO NPs, and 2) ZnO NPs core with an amorphous and heterogeneously distributed precipitated Zn_3_(PO_4_)_2_ shell (ZnO_Ph NPs). Both ZnO-based NPs were synthesized with ^68^Zn powder (Isoflex, USA) (99% ^68^Zn enriched). Both ZnO NPs and ZnO_Ph NPs synthesis were adapted from Dybowska *et al.*,^39^ Wu *et al.*,^40^ Rathnayake *et al.*^41^ and Muthukumaran and Gopalakrishnan.^42^ Detailed synthesis and characterization methods of these NPs were previously reported by Rodrigues *et al.*^18^ and more details can be found in the ESI.† Characterization included transmission electron microscopy (TEM), energy dispersive spectrometry (EDS), attenuated total reflection-Fourier transform infrared spectroscopy (ATR-FTIR), X-ray diffraction analysis (XRD), dynamic light scattering (DLS) and inductively coupled plasma mass spectrometry (ICP-MS).

### 2.2. Seed germination of pepper plants and growth conditions

Pepper seeds (*Capsicum annuum* L.) were obtained from Johnny's Selected Seeds (https://www.johnnyseeds.com/). Both seed germination and growth conditions were used exactly as in our previous study as follows.^18^ The seeds were soaked in deionized water (DIW) overnight, surface-sterilized in a 5% v/v bleach solution and then rinsed with DIW to remove all traces of bleach. Seeds were placed on DIW moistened towel paper in a Petri dish, kept in a growth chamber with a light/dark cycle of 16 h/8 h (25 °C/21 °C and 60% humidity) during 7 days for germination. Pepper seedlings were transferred into a 60 mL syringe filled with silica sand (ACROS Organics™). Pepper plants were used in this study as a model plant as they can produce fruits within 6 weeks under controlled growth chamber conditions. The silica sand washing method to remove all traces of metal and organic contamination was performed as previously reported by Rodrigues *et al.*, the details can be found in ESI.†^18^ A rope made of 100% cotton was introduced to connect the sand in the syringe to a ¼ strength Zn-free Hoagland solution (Table S2†), below the syringe, to maintain the sand humidity over time through capillary exchange (Fig. S1†). Plants were cultivated in the growth chamber under identical environmental conditions as for the seed germination described earlier, for the entire experiment (12 weeks).

### 2.3. Application of Zn-based treatments to the roots of pepper plants and plant harvesting

Three plants per treatment were exposed on the 6th week of growth and harvested at two timepoints: one week and six weeks (fruiting stage) after exposure. Plants were exposed to the treatments by using a micropipette to apply the materials directly in the sand next to the root. A total of 40 μg of ^68^Zn was added by applying 270 μL of 150 mg ^68^Zn per L of either ZnO NPs, ZnO_Ph NPs suspensions, or Zn ions solution. All NPs suspensions were prepared in ultrapure, Milli-Q (MQ) water. The dose applied here has been previously used to provide sufficient Zn for enabling plant growth while avoiding toxicity to pepper plants.^18,43,44^ Plants for all treatments grew similarly up to the fruiting stage, suggesting that the different treatments did not affect the physiological status of plants differently (Table S3†). No additional phenotype measurements were performed. The Zn control used in this study (Zn ions) consisted in preparing a ZnO NPs suspension of 150 mg ^68^Zn per L and acidifying it with 1 M HCl (Merck, Germany) solution until reaching pH 2 during 24 h to allow complete dissolution of the NPs and then using a 1 M NaOH (Panreac Química S.L.U., Spain) solution to increase to pH 7. Control plants were non-exposed plants, spiked with DIW and grown in the exact same conditions as the exposed ones. Plants were harvested and separated into leaves, stem, roots, and fruits (only for the six weeks after exposure). The roots were gently shaken to remove the adhered sand. The total duration of the experiment was 12 weeks, since exposure was performed at the 6th week of growth and the oldest plants (fruiting stage) were harvested six weeks after exposure.

### 2.4. Colloidal stability and ionic ^68^Zn release from ZnO NP and ZnO_Ph NP in Hoagland solution

The dissolution of ZnO NP and ZnO_Ph NP was assessed in the same Zn-free ¼ Hoagland solution as the one used for plant growth, at two timepoints (one week and six weeks). The zeta potential of the suspensions prior to centrifugation was measured by using a Zetasizer Nano-ZS90 (Malvern Instruments, UK), an average of 3 readings per sample were measured at each timepoint (Table 1). The dissolution test was performed as per in our previous study,^18^ by preparing 3 mg Zn per L suspensions of both ZnO-based NPs in Zn-free Hoagland solution and centrifuging an aliquot at 16 392*g* (Eppendorf® 5415R, rotor: F-45-24-11). The supernatant was then diluted with Milli-Q water (MQ water), acidified at 1% v/v HNO_3_ and analyzed by ICP-MS. The concentration used here was chosen to avoid saturation with Zn ions from the dissolution, but still be able to measure the dissolved Zn. More details regarding the method can be found in the ESI.†

**Table 1:** Zeta potential of ZnO NPs and ZnO_Ph NPs and their dissolution in Zn-free Hoagland's solution, after one week

	Time	Zeta potential^a^ (mV)	^68^Zn^2+^ release^a^ (mg ^68^Zn per L)	^68^Zn^2+^^a^ (%)	pH of the medium^a^
ZnO NPs	1 week	−3.9 ± 1.6	**0.82 ± 0.04**	**27 ± 1.33**	**5.73 ± 0.02**
	6 weeks	**−6.9 ± 0.05**	1.1 ± 0.01	37 ± 0.43	5.97 ± 0.01
ZnO_Ph NPs	1 week	−4.5 ± 0.7	**0.32 ± 0.01**	**11 ± 0.33**	**6.07 ± 0.02**
	6 weeks	−12 ± 1.3	1.3 ± 0.02	38 ± 0.72	5.98 ± 0.01

a The results are presented as mean ± standard deviation (*N* = 10 for zeta potential; *N* = 3 for ^68^Zn^2+^, P% and pH). N/A – not applicable. Zn-free Hoagland solution <0.03 mg ^68^Zn per L, pH 5.18 ± 0.01 and ionic strength 7.15 mM. The ZnO_Ph NPs contained 2.0 ± 0.1 wt% P. The bolded values are statistically significantly different between particles (*p* < 0.05).

### 2.5. Microwave digestion of pepper plant tissues for ICP-MS analysis

All plant tissues were oven-dried at 60 °C for 48 h, prior to microwave acidic digestion. All tissues of three plants per condition were acid digested by adding 70% v/v HNO_3_ (Merck, Germany) and 30% v/v H_2_O_2_ (Panreac Química S.L.U., Spain) to the dried samples overnight (∼12 h), then digested in a microwave oven (Table S4†). After cooling down, 37% v/v HCl (Merck, Germany) was added, samples were again digested in a microwave oven, obtaining a completely digested sample (clear solution with no precipitates). All digestates were diluted to 1% HNO_3_ (Merck, Germany) before being analyzed by ICP-MS (Agilent 7700). The detailed digestion protocol was used as reported in our previous study and can be found in the ESI.†^18^

### 2.6. Zn distribution and speciation on pepper fresh tissues using micro X-ray fluorescence (μ-XRF) and micro X-ray absorption near-edge structure (μ-XANES)

The scanning X-ray microscope at the ID21 beamline of the European Synchrotron Radiation Facilities (ESRF, Grenoble-Fr) was used to perform μ-XRF maps and μ-XANES measurements under cryo-conditions.^45^ Fresh exposed roots and stems were embedded in optimal cutting temperature resin (OCT) (Sakura Finetek, USA) and flash-frozen in liquid nitrogen. Cross-sections of 20 μm thick were done under cryogenic conditions (−50 °C) using a cryo-microtome (Leica RM2265, LN22) at the beamline and cross-sections were transferred, still frozen, on the cryostage of the beamline. μ-XRF maps were performed at 9.8 keV using a ∼0.5 × 0.7 μm spot size and a 100 ms dwell time. μXANES spectra at Zn K-edge were collected on points of interests (POIs; Zn hotspots) from 9.65 keV to 9.80 keV using a 0.5 eV energy step and 100 ms dwell time. Synthesis and sample preparation of all compounds used as XANES references were performed as in our previous study^18^ and can be found in ESI† (Table S5). The respective averaged XANES spectra obtained for each reference can be found in Fig. S2 in ESI.† More details regarding sample preparation can be found in ESI.†

PyMCA software (version 5.8.1) was used for deadtime correction, intensity normalization and fitting of the X-ray fluorescence spectra.^46^ The elemental distribution maps obtained were overlayed as RGB images. Signal intensity profiles for Zn were selected on the corrected μXRF maps. A profile represents the average of the Zn fluorescence intensity measured on each pixel from all the rows on a given column in a selected part of the μXRF map. For that purpose, three rectangles were drawn per μXRF map and averaged between them to represent the Zn profile intensity on roots and stems for all treatments (Fig. S3†). Elemental distribution maps of the control DIW plants can be found in ESI† (Fig. S4).

XANES spectra were analyzed using orange data mining software (version 3.36.2) with the spectroscopy add-on.^47,48^ XANES spectra were converted into second derivative, averaging 19 floating points to reduce for noise contribution to the signal, and principal component analysis (PCA) was performed on the intensity of the vector-normalized second derivative at each energy step (0.5 keV).^49^ Average XANES spectra of representative groups obtained from the PCA were further exported. Spectra normalization and linear combination fitting (LCF) were performed using larch software (version 0.9.72).^50^ The number of POIs where μ-XANES spectra were collected and the LCF results obtained from the fitting of the μ-XANES spectra in the exposed roots and stem one week after exposure are shown in Table S7.†

### 2.7. Statistical analysis

The data were analyzed using IBM SPSS Statistics (Version 29.0). Significant statistical differences between total uptake and translocation of 68Zn were assessed using one-way ANOVA analysis (*p* < 0.05 threshold) between the different Zn treatments.

## 3. Results and discussion

### 3.1. Nanoparticle characterization and dissolution

Detailed characterization of these ZnO-based NPs were previously described by Rodrigues *et al.*^18^ The primary particle size of ZnO NPs (26 ± 8 nm) and ZnO_Ph NPs (48 ± 12 nm) was determined by TEM (Fig. S5 and S6†). Other NP properties such as zeta potential and hydrodynamic diameter measured in MQ water can be found in ESI† (Table S6). The presence of a heterogeneous amorphous Zn_3_(PO_4_)_2_ layer at the ZnO_Ph NPs surface was previously verified by EDS and FT-IR and both ZnO-based NPs had a similar shape (polymorphic).^18^ Both ZnO-based NPs were negatively charged in Hoagland's solution (Table 1), despite the ZnO NPs being positively charged in MQ water (14.8 mV). Similar results were reported by Li *et al.*,^51^ in which ZnO NPs (regardless of size differences) suspended in Hoagland's solution were negatively charged.

The observed differences in dissolution between both ZnO-based NPs after 1 week (Table 1) could potentially be explained by the differences in pH. The *K*_sp_ of ZnO (Zn(OH)_2_) is 4.5 × 10^−17^ and is pH-dependent. The solubility should decrease as the pH increases. The Zn_3_(PO_4_)_2_ shell on ZnO NPs has a relatively low solubility at pH 6 (*K*_sp_ ≈ 9.1 × 10^−33^)^41^ which could also be lowering the rate of dissolution of the ZnO NP core. The presence of the orthophosphate groups (from KH_2_PO_4_) in the Hoagland's solution (0.25 mM KH_2_PO_4_) could also limit dissolution of the Zn_3_(PO_4_)_2_ shell. Rathnayake *et al.*^41^ demonstrated that after 7 days, the dissolved Zn concentration of ZnO particles was substantially lower in phosphate solution (1.05 mM of Na_2_HPO_4_) at pH 6 (<7.7 × 10^−5^ mM Zn) compared to solutions without phosphate (3.58 × 10^−4^ mM Zn). After 6 weeks, differences in dissolution between both ZnO-based NPs were no longer observed (37% dissolution for ZnO NPs and 38% for ZnO_Ph NPs), suggesting that the two particles had similar controls on their dissolution rates.

### 3.2. ^68^Zn root uptake and *in planta* translocation

**Fig. 1:**
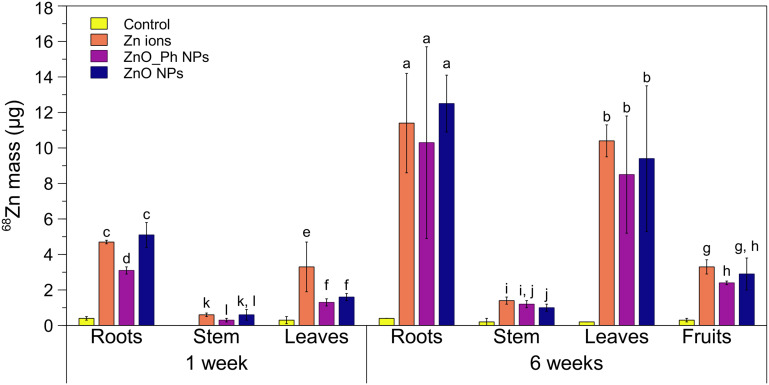
Mass of ^68^Zn (in μg) in the roots, stem, leaves and fruits of pepper plants, one week and six weeks after exposure to ZnO NPs, ZnO_Ph NPs and Zn ions (dissolved Zn^2+^_(aq)_). The control represented here comes from non-exposed pepper plants which have a low ^68^Zn signal due to naturally occurring ^68^Zn isotope (∼18%). Three plant replicates per treatment were used to calculate the means and standard deviations (presented as error bars). Statistically significant differences between the mean values for the treatments are represented with different letters (*p* < 0.05).

One week after exposure, there was significantly more ^68^Zn associated to the roots of pepper plants exposed to either Zn ions or ZnO NPs (12–13% of the initial dose applied) compared to ZnO_Ph NPs (7.7% of Zn applied) (Fig. 1 and S5†). The differences observed in root association between both ZnO-based NPs treatments cannot be related to their surface charge, since both have a similar (low) negatively charge in Hoagland solution (Table 1). The similarity of Zn uptake for ZnO-NPs and Zn^2+^ ions suggest that difference is related to the lower dissolution of the ZnO_Ph NPs compared to the ZnO NPs. The higher ZnO NPs dissolution provides more Zn^2+^ which associates with and moves into the roots for that treatment.^8,33^ Despite the lower amount of ZnO_Ph NPs associated with roots compared to ZnO NPs and lower solubility of ZnO_Ph NPs compared to ZnO NPs, there were no statistically significant differences for ^68^Zn mass translocation to the stem and leaves between these treatments. Furthermore, the amount of Zn mass translocated from roots to both stem and leaves was higher for the ZnO_Ph NPs treatment than for ZnO NPs (0.52 for ZnO_Ph NPs *vs.* 0.43 for ZnO NPs). This suggests that the lower dissolution of ZnO_Ph NPs promoted by the Zn_3_(PO_4_)_2_ shell led to lower available Zn^2+^ in that treatment. This could have enhanced Zn translocation to upper tissues using different Zn transport mechanisms compared to ZnO NPs treatments. This hypothesis is confirmed later in the paper using synchrotron XRF mapping of the tissues. It is also noteworthy that both ZnO-based NPs resulted in less total uptake and translocation to stems and leaves compared to Zn^2+^ ions after 1 week.

The amount of ^68^Zn root association increased up to 3-fold after 6 weeks of exposure compared to 1 week after exposure, which was likely due to the increase in water and nutrient intake during plant growth as well as continued NP dissolution. The longer time also eliminated any statistically significant differences in ^68^Zn association to the roots observed after 1 week (Fig. 1). The similar association of ^68^Zn with plant roots for both NPs treatments is consistent with their similar dissolution amounts after 6 weeks. However, there was also no statistically significant difference between the Zn-based NPs when compared to the Zn ion control, which is 100% dissolved Zn^2+^. This suggests that the root system might have reached a maximum for Zn association in the Zn ion treatment. Between 12 and 13 μg of ^68^Zn (30–33% of the initial applied Zn NP masses) was translocated to the stem, leaves, and fruits with no statistically significant differences between ZnO_Ph NPs and ZnO NPs treatments. Between 2.4–2.9 μg (6% to 7.3%) of ^68^Zn total NP mass applied to roots reached the fruits. This represents 10.7–11.2% of the Zn mass that was taken up by the plant roots. These values are not statistically significantly different that than observed for the applied Zn^2+^ ions.

### 3.3. Zn cellular distribution

**Fig. 2:**
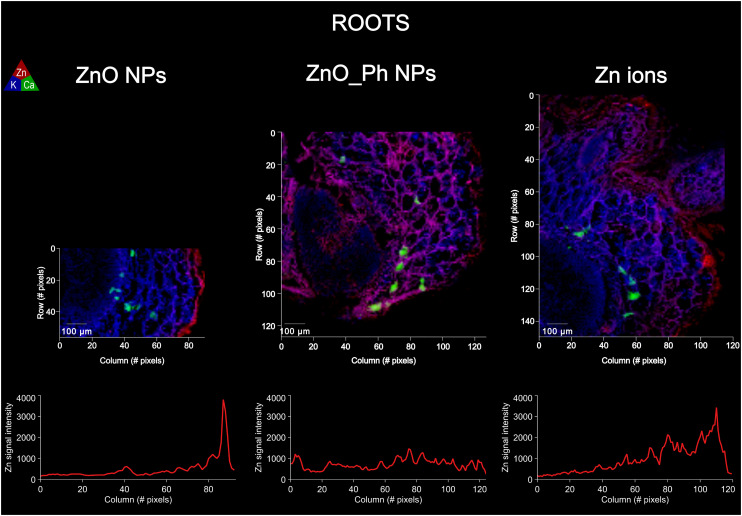
Elemental μ-XRF maps on roots of pepper plants exposed to ZnO NPs, ZnO_Ph NPs and Zn ions: one week after exposure. The Kα fluorescence of Zn is represented in red, K in blue and Ca in green. Bellow each μ-XRF map is the average fluorescence intensity measured for Zn along a selected root cross-section. The averaging of Zn Kα fluorescence intensity was performed by averaging 3 profiles per μ-XRF map (Fig. S3†).

**Fig. 3:**
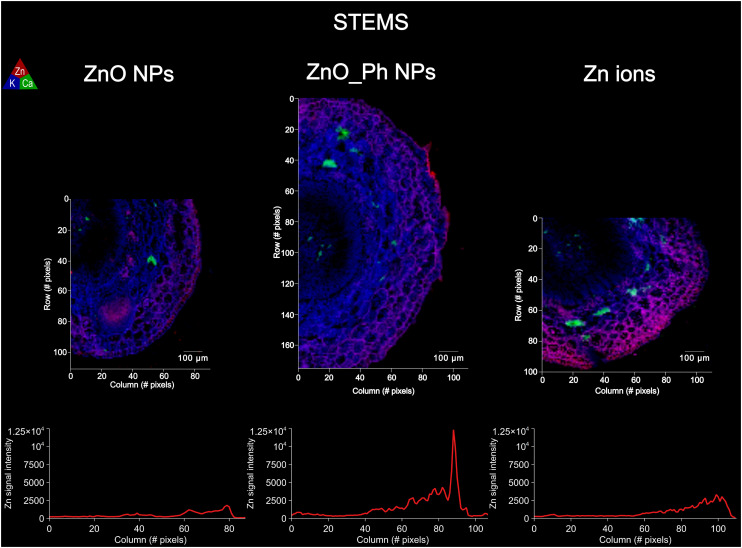
Elemental μ-XRF maps on the stems of pepper plants exposed to ZnO NPs, ZnO_Ph NPs and Zn ions: one week after exposure. The Kα fluorescence of Zn is represented in red, K in blue and Ca in green. Bellow each μ-XRF map is the average fluorescence intensity for Zn along a selected cross-section. The averaging of Zn Kα fluorescence intensity was performed by averaging 3 profiles per μ-XRF map (Fig. S3†).

Zn fluorescence intensity profiles from synchrotron μ-XRF maps within roots and stems cross-sections of similar thickness were used as a proxy for Zn accumulation in plant tissues (epidermis, cortex and vasculature) between treatments (Fig. 2 and 3). The association of Zn to other elements in those tissues for each treatment was also assessed by Pearson correlation (Fig. S9–S14†).

The Zn fluorescence profiles within the root tissues of pepper plants shows that Zn concentration and distribution in the epidermis, cortex and vasculature varied depending on the treatment. The Zn fluorescence intensity in the roots of pepper plants exposed to Zn ions or ZnO NPs showed that majority of Zn was either adhered to the outside or within the root epidermis. Despite the similarities in Zn epidermis accumulation between the Zn ions and ZnO NPs treatments, the Zn profiles suggest that the Zn ions were more readily translocated to the root cortex. This is consistent with the incomplete dissolution of the ZnO NPs resulting in less Zn^2+^ ions for translocation into the cortex.

The ZnO_Ph NPs exposed plants had lower Zn association to the epidermis than for the ZnO NPs and Zn ions treatments, which is consistent with the lower dissolution rate and lower amount of Zn^2+^ ions available for uptake. However, the Zn profile intensity within the root cortex towards vasculature for the ZnO_Ph NPs treatment was higher and more evenly distributed inside the root than for the other two treatments. This suggests that the lower Zn^2+^ available for uptake in the ZnO_Ph NPs exposed plants triggered a different route for Zn transport within the root cortex and towards the vasculature. It is possible that the higher Zn deficiency in the growth medium for the ZnO_Ph NPs treatment induced a shift in the metabolomic translocation of Zn towards the vasculature and upper tissues. Our previous study similarly showed higher Zn mobility for foliar applied ZnO_Ph NPs compared to ZnO NPs.^18^

The differences observed in Zn cellular distribution within the roots were also associated with co-location of Zn with other elements in that tissue. We observed that Zn in roots was highly co-localized with K, Cl, and S and moderately co-localized with P for the ZnO_Ph NPs treatment, while this was not observed for ZnO NPs and Zn^2+^ ions (as shown with Pearson correlations Fig. S9–S11†). This co-association difference indicates that the lower bioavailable Zn^2+^ for the ZnO_Ph NPs treatments lead to a higher P root uptake from the plant growth medium and consequently to a different Zn and P distribution in plant cells. Previous studies have shown that plants grown in a Zn deficient medium lose some ability to regulate Pi-related genes and phosphate homeostasis, increasing phosphate accumulation in leaves.^26,52–55^ Indeed, Zn derived from the ZnO_Ph NPs was associated with membranes of cells and organelles (see for instance Fig. S15†), while Zn from ZnO remains associated with the epidermis cells (likely in their cell walls). Several hypotheses could explain this result: (i) an increased ZnO dissolution at the root surface, triggering immobilization of Zn within the epidermis cell walls, and/or (ii) an increased Zn and P mobility due to the lower Zn^2+^ uptake and Pi-related genes activation.^30,52^

Regarding the distribution in the stem, all treatments led to a similar Zn distribution, with a higher amount of Zn in the epidermis (outer cell layer) compared to the vasculature (inside) (Fig. 3). However, the highest Zn fluorescence intensity was detected in the stem epidermis of plants exposed to ZnO_Ph NPs. Our results suggest that regardless of the form of Zn applied to the roots, or the concentration reaching the stem, Zn is transported and preferentially accumulates in the stem epidermis. Also, we observed that for all treatments the Zn in the stem correlated and co-localized to S, Cl and K (Fig. S12–S14†). This result suggests that once Zn has been translocated from the root to upper parts of the plant, the mechanisms of cellular translocation become similar among these treatments.

### 3.4. Zn *in planta* biotransformation and mobility following root uptake

To better understand the differences in Zn distributions between the treatments (Fig. 2 and 3), Zn speciation was determined at selected points of interest (POIs) in the epidermis, cortex, and vasculature of exposed roots and stems. POIs were Zn hotspots identified in those tissues by synchrotron μ-XRF mapping. It should first be noted that there was no evidence of ZnO NP or ZnO_Ph NP present in either roots or stems of NP-exposed plants (Fig. S16 and S17†), suggesting that it is primarily ionic Zn entering the roots for all treatments (see linear combination fitting results Table S7†).

**Fig. 4:**
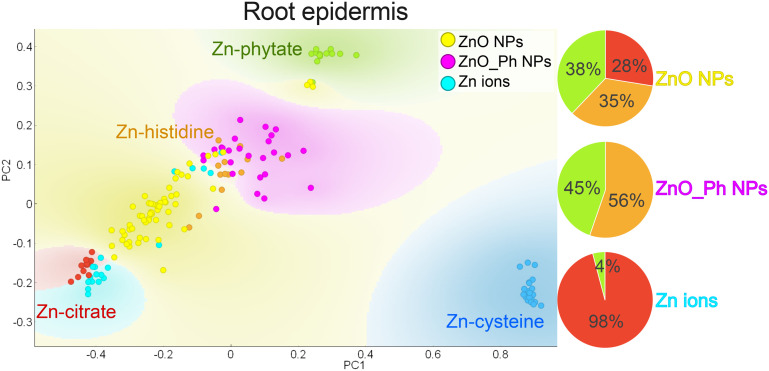
PCA of XANES spectra collected on selected points of interest (POIs) in the exposed root epidermis of pepper plants exposed to ZnO_Ph NPs, ZnO NPs or Zn ions one week after exposure. The following references were used for comparison: Zn-cysteine in blue (Zn-thiol), Zn-citrate in red (Zn-carboxyl), Zn-phytate in green (Zn-phosphate) and Zn-histidine in orange (Zn-carboxyl). Eigenvalue PC1: 0.80 and PC2: 0.15.

We discuss the Zn speciation in the plant samples along its path from the point of entry (root epidermis) to the stem (Fig. S18†). This includes, the root epidermis, the root cortex, the root vasculature, the stem vasculature, the stem cortex and, finally the stem epidermis. PCA for POIs obtained from the root epidermis was different between all the treatments (see PCA Fig. 4), suggesting that the plants responded to each form of Zn differently. Plants exposed to ionic Zn mainly clustered near Zn-citrate and plants exposed to ZnO NPs near Zn-citrate, Zn-histidine and to some extent Zn-phytate. These results indicate that for both ionic Zn and ZnO NPs treatments Zn was primarily associated with carboxyl groups (Zn-citrate and Zn-histidine references). Cell walls contain proteins rich in carboxyl groups, with a high affinity for Zn.^56^ Zn-Carboxyl binding has been observed in the cell walls of leaves and in the stems of pepper plants.^18^ The higher Zn accumulation in the root epidermis discussed in the previous sections (see Table 1 and Fig. 2) together with the high incidence of carboxyl binding for both ZnO NPs and ionic Zn treatments strongly suggests that the applied Zn ions and ZnO NPs are mainly taken up as Zn^2+^ and supports the hypothesis that the Zn taken up for those treatments accumulates within the epidermis cell wall.

In contrast, Zn in the root epidermis of plants dosed with ZnO_Ph NPs clustered near Zn-histidine and Zn-phytate references. The ZnO_Ph treatment had a lower Zn association to roots compared to Zn ions and ZnO NPs (see Fig. 1), but it was more uniformly distributed within the root cross section (see Fig. 2) and had a high co-localization with K (Fig. S11†) (symplastic space and organelles association). Given that plants in our study were grown in a medium without Zn, up to exposure, and that Zn^2+^ provided by the ZnO_Ph NPs was lower than for the other treatments Pi-gene activation of different protein transporters may have been triggered,^26,27,53,54^ moving Zn more efficiently towards the vasculature compared to the other treatments. This is also consistent with the higher Zn-phytate like nature of the Zn speciation. However, this study did not assess if the pepper plants were in fact Zn deficient, the difference in the Zn-phytate like species in between treatments is minor, and Zn and P transporters were not measured. Thus, these must be further confirmed in the future.

**Fig. 5:**
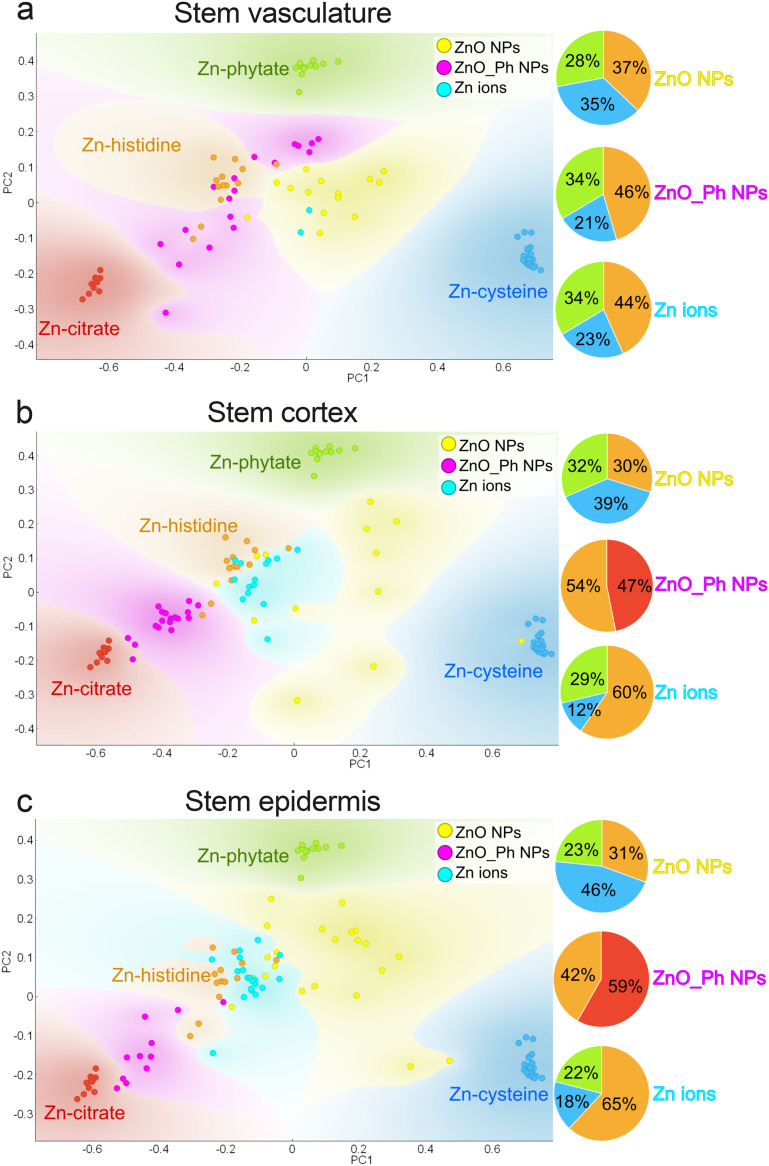
PCA of XANES collected on selected POIs in the stem (a) vasculature, (b) cortex and (c) epidermis of pepper plants exposed to ZnO_Ph NPs, ZnO NPs or Zn ions 1 week after exposure. The following references were used for comparison: Zn-cysteine in blue (Zn-thiol), Zn-citrate in red (Zn-carboxyl), Zn-phytate in green (Zn-phosphate) and Zn-histidine in orange (Zn-carboxyl). Eigenvalue for (a) PC1: 0.77 and PC2: 0.16, (b) PC1: 0.79 and PC2: 0.15 and (c) PC1: 0.77 and PC2: 0.16.

From the epidermis, Zn moves into the root cortex and vasculature, then into the stem vasculature. The studied POIs in the root cortex and vasculature had a similar average Zn speciation for all treatments, with Zn bound to carboxyl and phosphate groups (Fig. S19 and S20†). All three Zn treatments also had similar speciation in the stem vasculature, but also included a thiol-like Zn species (Zn-cysteine reference) (Fig. 5a). This additional Zn association to thiol groups in the stems suggests that Zn associates either with metal tolerance proteins, or transporters, that are rich in thiol groups.^5^ This is an indicator that Zn is either being sequestrated, transported and/or distributed for plant growth or could be a plant response to Zn toxicity.^5^ These results suggest that Zn transport mechanisms in those tissues are similar for all three treatments.

Zinc in the stem vasculature can move into the stem cortex and epidermis. The Zn derived from the ZnO_Ph NPs treatment clustered differently from the other two treatments (see Fig. 5b and c). Zinc in the stem cortex and epidermis was mainly associated to carboxyl groups (histidine and citrate model compounds) for the ZnO_Ph NPs treatments. In contrast, ZnO NPs and ionic Zn exposure led to Zn-phytate, Zn-histidine, and Zn-cysteine-like species. This difference in Zn transport and accumulation in the stem epidermal cells may again be due to the lower Zn uptake (see Fig. S15†).^18,57^ The lower amount of Zn and the presence of Pi is immobilizing Zn in the stem epidermis as a storage mechanism for later use during plant growth.

### 3.5. Evaluation of application strategies in Zn uptake and transport mechanisms

Our previous published study applied the same types and massed of Zn-based NPs to pepper plants, but used foliar application rather than root application.^18^ This provides a unique opportunity to compare the uptake, translocation, and speciation of Zn in plants depending on the type of application, *i.e.* root *vs.* foliar, and the resulting environmental implications of those differences.

**Fig. 6:**
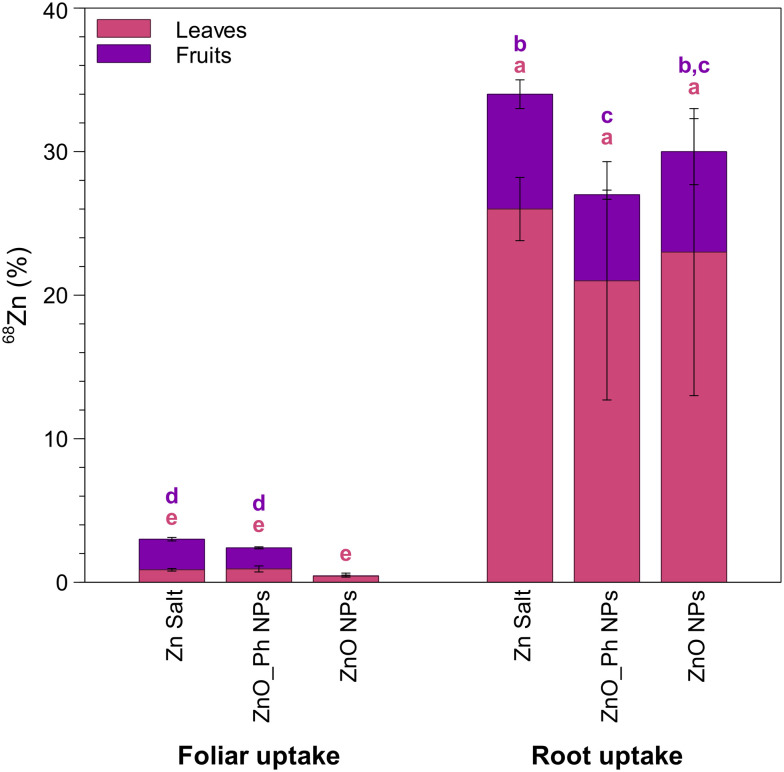
Translocation of ^68^Zn (in % relative to the initial dose applied 40 μg Zn per plant) to the non-exposed leaves and fruits of pepper plants, 6 weeks after foliar or root exposure to ZnO NPs, ZnO_Ph NPs and Zn ions. Error bars represent the weighted standard deviation of the samples from three replicate plants. The different letters on top of each bar chart indicate statistically significant differences (*p* < 0.05). Letters in purple indicate statistically significant differences of the means for ^68^Zn mass translocated to fruits between treatments. Letters in pink indicate statistically significant differences of the means for ^68^Zn mass translocated to leaves between treatments.

Foliar and root application resulted in different efficiencies of use, with root application providing significantly higher Zn translocation to leaves and fruits. Foliar application translocated only 2.4% of the total applied Zn for ZnO_Ph NPs and 0.5% of the total applied Zn for ZnO NPs to leaves and fruits (Fig. 6). In contrast, 27% and 30% of the applied Zn translocated to leaves and fruits for ZnO_Ph NPs and for ZnO NPs, respectively, when Zn was root applied (Fig. 6). For foliar applications, only ZnO_Ph NPs promoted Zn translocation to the fruits (1.5% of the total applied Zn). For root applications, both ZnO_Ph NPs and ZnO NPs delivered Zn to the fruits (6.0% and 7.3% of the total applied Zn respectively). Foliar fertilization has been described as a more efficient approach to deliver nutrients to specific plants tissues compared to root uptake because it reduces losses to soil minerals or from leaching.^58–61^ Nevertheless, in our study, root application more efficiently delivered Zn into pepper plants (65% for ZnO NPs and 56% for ZnO_Ph NPs) than foliar application (1.8% for ZnO NPs and 5.8% for ZnO_Ph NPs) (Fig. S21†). However, the efficiency of Zn translocation to the fruits after Zn uptake was higher in the case of foliar applied Zn. For the foliar exposure, ∼27% of the total Zn that was taken up translocated to the fruits for ZnO_Ph NPs, while for the root exposure, only ∼10% of the total Zn taken up was translocated to the fruits. While no Zn translocation to the fruit was observed for the foliar exposure to ZnO NPs, when root exposed, ∼11% of the total Zn taken up was translocated to the fruit. Despite the higher Zn translocation to the fruit for the foliar exposure, the root exposure was overall more efficient for total Zn uptake inside the plant. One should also note that the plants in the present study were grown in sand and the NPs suspensions were pipetted near the roots, so these differences may be less significant in real soils even if using precision drip irrigation.

Depending on the application location (foliar or root), there are different mechanisms for Zn uptake and transport involved, which led to different plant strategies for Zn storage and transport. The first main difference is that NP uptake and translocation was only observed for the foliar exposure, not for the root exposure. This difference was likely due to the fact that NP do not dissolve as readily at the leaf surface as at the root surface because there is less water at the leaf surface and higher chance of localized Zn ions saturation limiting dissolution. It is also worth mentioning that NPs can cross the leaf surface, for example through stomata,^31^ and no barrier will prevent NPs from reaching the leaf vasculature and translocating to other plant tissues. For roots however, even if NPs were to be taken up, the Casparian strip would likely prevent NPs to reach the vasculature, preventing its translocation to other plant tissues. The differences in Zn speciation in the stem vasculature when ZnO-based NPs were either root or leaf applied further indicated that Zn was being transported differently. For instance, for the leaf application, both ZnO-based NPs were only associated to carboxyl and phosphate groups, while for the root application Zn was also found associated to thiol groups. This additional association to thiol groups in the stem vasculature may have contributed to reduce Zn mobility towards the fruits for root *vs.* foliar applied ZnO-based NPs. These results highlight the importance of understanding the plant physiological responses when using different forms of NPs or when using different routes of application of the particles, and its implications for Zn-fertilizer optimization in agricultural systems.

In both studies, factors that likely affected the foliar Zn delivery efficacy when compared to the root application were: (i) foliar exposed plants lost (dropped) the exposed leaves after 1 week of exposure, which prevented plants from taking up more Zn after 1 week; (ii) the amount of Zn that was not washed-off the exposed leaves after 1 week was ∼20%, and the dissolution in MQ water after 1 week was ∼20% for both ZnO-based NPs, so the Zn pool available for uptake was only ∼4% of the initial foliar applied dose, (iii) the dissolution of the NPs in the Hoagland's solution applied to the roots reached 37% for ZnO NPs and 38% for ZnO_Ph NPs after 6 weeks, which provided a continuous Zn supply to the roots up to the fruiting stage. This suggest that foliar Zn delivery efficacy could be further improved by improving the rainfastness of the particles controlling the dissolution of Zn-based NPs. In both studies, plants were exposed at the 6-week growth stage, however it would be important to assess whether foliar uptake of Zn would be improved when applying these treatments at earlier, later or at several stages in the plant's growth, or to more leaves, and, if by doing so, foliar application could then be more efficient in fortifying the plants and ultimately the fruits. Our results highlight the need to further study how to maximize Zn uptake efficiency for both application strategies, in particular the foliar application, while minimizing the possible environmental impacts from Zn losses.

### 3.6. Environmental implications

Our findings suggest that Zn uptake by the roots occurred primarily as dissolved Zn^2+^ after the dissolution of NPs at the root surface or in the rhizosphere. Although uptake of a nanoform was not observed, it cannot be completely ruled out. Regardless, Zn uptake, translocation, cellular distribution, and storage strategies for the ZnO_Ph NPs treatment were clearly different than for the Zn ions treatment. After 1 week, the Zn-phosphate shell lowered ZnO_Ph NPs dissolution and promoted Zn movement towards the root vasculature and shifted Zn storage mechanisms towards the stem epidermis cell walls (carboxyl binding), likely by enabling the upregulation of specific genes that promoted Zn uptake and translocation mechanisms through different protein transporters. On the contrary, for both Zn ions and ZnO NPs treatments, Zn was mostly stored in the root epidermis cell walls and was more mobile in the stem (phytate binding). These results imply the potential to improve the efficiency of NP-based fertilizers by taking advantage of the plant's physiological response to the material.

The current challenges faced regarding the use of agrochemicals to increase food security, makes it a priority to develop new fertilizers that can enhance both agricultural sustainability and food nutritional value. This study highlights the potential of designing nanomaterials made from mixtures of micro- and macronutrients (*e.g.* through a phosphate shell) to stimulate root uptake and manipulate cellular distribution in view of delivering Zn and other nutrients to plants in one application. Delivering two essential micronutrients (in this case P and Zn) to crops in only one application appears to improve the Zn distribution towards edible plant parts. In the foliar application, the Zn-phosphate shell led to ∼27% of the Zn that was taken up by the plant being translocated to the fruits. If the foliar uptake efficiency of Zn-based nanofertilizers could be increased, it could be a good method to fortify foods with nutrients while reducing the amount of Zn-based fertilizers that are applied each year to soils to maintain an adequate Zn nutritional status of crop plant fruits and grains. Despite the lower Zn translocation efficiency to the fruits when root applied (∼10–11% of the total Zn that was taken up by the plant), this application strategy is overall more efficient at loading Zn into the plants. Better control over the NP dissolution rate, for example by thickening the Zn-phosphate shell, could improve the uptake efficiency and Zn delivery to the fruits. This would be an interesting strategy to improve fertilization and utilization efficiency of soil-applied micronutrients to lower the environmental impacts of agriculture, save mineral resources, reduce costs of application but more importantly to safely improve the nutritional value of crops.

## Conflicts of interest

There are no conflicts to declare.

## Supplementary Material

EN-012-D5EN00217F-s001

## Data Availability

The data supporting this article have been included as part of the ESI.†
