# Supplementary material for: Zn3(PO4)2 shell effects on Zn uptake and cellular distribution of root applied ZnO NPs
Source: Environ Sci Nano. 2025 May 29;12(7):3639–52. doi: 10.1039/d5en00217f (PMC12121551; doi:10.1039/d5en00217f)
Supplement: EN-012-D5EN00217F-s001 [file EN-012-D5EN00217F-s001.pdf]

Supplementary Information (SI) for Environmental Science: Nano.
This journal is © The Royal Society of Chemistry 2025

Supporting Information

$Zn_3(PO_4)_2$ shell effects on Zn uptake and cellular distribution of root applied ZnO NPs

Sandra Rodrigues [a *], Astrid Avellan [b], Hiram Castillo-Michel [c], Matheus C.R. Miranda [d], Diana Salvador [e], Aude Calas [b], Gregory V. Lowry [g], Sónia M. Rodrigues [a].

[a] Centre for Environmental and Marine Studies (CESAM), Department of Environment and Planning, Universidade de Aveiro, 3810-193, Aveiro, Portugal

[b] Géosciences Environnement Toulouse - CNRS- IRD- CNES- Université Toulouse III- Observatoire Midi-Pyrénées, 31400 Toulouse, France

[c] ESRF, The European Synchrotron, 71 Avenue des Martyrs, CS40220, 38043 Grenoble Cedex 9, France

[d] Centre for Environmental and Marine Studies (CESAM), Department of Chemistry, Universidade de Aveiro, 3810-193, Aveiro, Portugal

[e] Centre for Environmental and Marine Studies (CESAM), Department of Biology, Universidade de Aveiro, 3810-193, Aveiro, Portugal

[g] Department of Civil and Environmental Engineering, Carnegie Mellon University, Pittsburgh, Pennsylvania 15213, United States

* Corresponding author. E-mail address: sandra.rodrigues@ua.pt

Number of pages: 21

Number of tables: 7

Number of figures: 21

2. Materials and methods

2.1. ^{68}ZnO-based NP syntheses and characterization

Synthesis

Summarily, ZnO NPs were produced by obtaining Zn acetate, dissolving metallic ^{68}Zn powder in acetic acid (Sigma Aldrich) at 80 °C under stirring for 30 h. The white powder precipitate that was formed was then dried at 50 °C for 24 h. The Zn acetate was then reduced with methanol (Fisher Scientific, UK) and refluxed at 65 ºC. Water was added to the previous solution and then a methanol solution containing NaOH (Panreac Química S.L.U., Spain) was added dropwise until the obtention of a white precipitate. The produced ZnO NPs powder was then retrieved through centrifugation and dried in a desiccator at room temperature. The ZnO_Ph NPs were synthesized by dispersing the previously produced ZnO NPs in a Na_2HPO_4 (Merck, Germany) solution (pH 8) for 72h. The suspension was then centrifuged at 4000g for 1h and washed with Milli-Q water (MQ water) twice (no dissolved Zn was detected on the supernatant by ICP-MS analysis). The resulting powder was then dried at room temperature on a desiccator.[1]

Characterization

Nanoparticle images were obtained by Transmission Electron Microscope (TEM), using a Hitachi HT22700B coupled to a dispersive energy spectrometer (EDS) (electron accelerating voltage of 200 kV). Sample preparation involved dispersing the nanoparticles in milli-Q water and depositing them on gold grids of carbon film. Nanoparticle sizes were evaluated using the ImageJ software. The average particle size was obtained by measuring the size of 150 particles.

Surface charge was determined at different pH by using a Zetasizer Nano-ZS90 (Malvern Instruments, UK). An average of 10 readings per sample were measured.

Attenuated total reflection-Fourier transform infrared spectroscopy (ATR-FTIR) was used to analyze the surface chemistry of the nanoparticles. Measurements were performed using an Avatar 360 Thermo Nicolet spectrometer equipped with a diamond ATR window. Samples were scanned in transmission mode over the range of 400–4000 cm^{-1} with a resolution of 4 cm^{-1}. Each spectrum represents an average of 64 scans.[1]

X-ray diffraction analysis (XRD) was conducted using an Empyrean diffractometer (PANanlytical, The Netherlands) with Cu-Kα radiation. The diffraction patterns were obtained using a step scan program with 0.02° per step and a 5 second acquisition time over a range of 10 to 60°. XRD data were processed using Match 3 (PANanlytical BV Almelo, The Netherlands) for the identification of crystalline phases in the samples.[1]

The zinc content analysis was performed using an Inductively Coupled Plasma Mass Spectrometry (ICP-MS, Thermo-X Series) on an Agilent 7700x ICP-MS. The following protocol for quality control was performed as follows:

A calibration curve of 0, 10, 30, 50, 100, 500 and 1000 µg/L was established using calibration standards for Zn determination. The curve was linear with an r^2 value of 0.999. An independent certified standard was used to verify the calibration accuracy. The method's precision was assessed by analyzing five replicate samples, with a relative standard deviation (RSD) ≤ 10%. The detection limits (DL) was determined to be 4 µg/L, calculated as three times the standard deviation of ten blank measurements.

Nanoparticle samples were digested in a microwave (Table S1) (Speedwave 4, Berghef) by mixing 1 mg of nanoparticles with 1.5 mL of HNO_3. The volume was made up to 25 mL of Milli-Q water and measurements were performed in triplicate.[1]

Table S1 - Digestion program used in the microwave oven for NPs.

	Temperature (ºC)	Pressure (psi)	Ramp (ºC /min)	Time (min)	Power (watts)
**1**	180	50	5	15	90
**2**	50	50	1	5	0
**3**	50	50	1	1	0
**4**	50	0	1	1	0
**5**	50	0	1	1	0

2.2. Seed germination of pepper plants and growth conditions

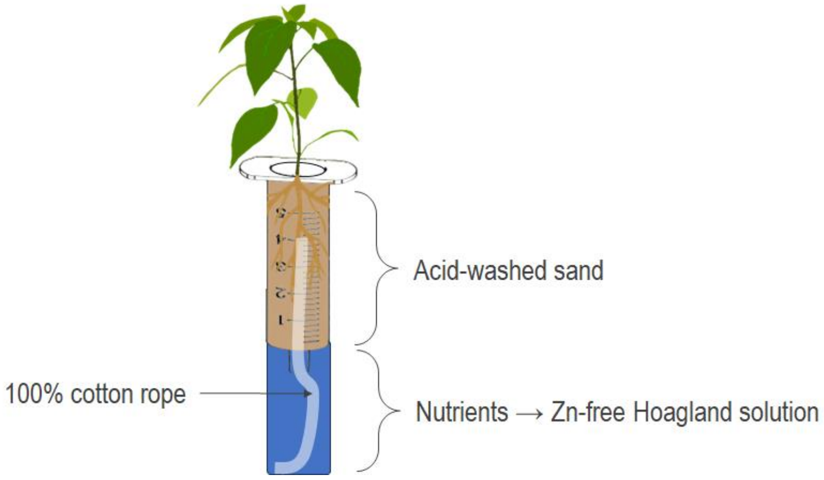

Figure S1 – Diagram of the plant cultivation setup.

Silica sand washing protocol:

The silica sand was previously washed with DIW, followed by acid-washing (5% v/v HNO_3) overnight, rinsed with DIW, dried at 90ºC for 24h (for water evaporation), then burned at 250ºC overnight to remove salicylic acid and finally rinsed thoroughly with DIW.[1]

Table S2 - Chemical composition of Zn-free ¼ strength Hoagland solution

Chemicals	Concentration (mM)
**KNO_3 (Fisher Scientific, UK)**	1.29
**$Ca(NO_3).4(H_20)$ (Fisher Scientific, UK)**	1.20
**$MgSO_4.7(H_2O)$ (Merck, Germany)**	0.50
**KH_2PO_4 (Merck, Germany)**	0.25
**Na(FeIII)-EDTA (Merck, Germany)**	5.00×10^{-3}
**H_3BO_3 (Merck, Germany)**	11.56×10^{-3}
**$MnCl_2$ (Merck, Germany)**	2.29×10^{-3}
**$Na_2MoO_4.2H_2O$ (Merck, Germany)**	0.12×10^{-3}
**$CuSO_4.5H_2O$ (Merck, Germany)**	0.05×10^{-3}

2.3. Application of Zn-based treatments to the roots of pepper plants and plant harvesting

Table S3 – Total plant dry biomass per treatment

Treatment	Dry mass (g)	
	1 week	6 weeks
**Zn ions**	0.48 ± 0.05	1.63 ± 0.57
**ZnO_Ph NPs**	0.48 ± 0.05	1.30 ± 0.23
**ZnO NPs**	0.58 ± 0.09	1.15 ± 0.28

2.4. Colloidal stability and ionic ^{68}Zn release from ZnO NP and ZnO_Ph NP in Hoagland solution

In the present study, 3 mg Zn L^{-1} suspensions of each nanomaterial was prepared in Zn-free Hoagland solution, in 50 mL tubes. The tubes were laid horizontally on a reciprocating shaker (150 rpm) in the dark for the entire duration of this test. Aliquots were taken from the tubes after 1 and 6 weeks and centrifuged for 30 min at 16,392 g

(Eppendorf® 5415R, rotor: F-45-24-11). The supernatant (the top 0.5 mL) was then diluted with MQ water, acidified at 2 % v/v HNO3 and analyzed by ICP-MS.[1]

2.5. Microwave digestion of pepper plant tissues for ICP-MS analysis

The ratio of sample used for digestion and acids was as follows: 25 – 100 mg sample d.w. : 0.5 mL HNO_3 : 0.25 mL H_2O_2 : 0.25 mL HCl. The digestion consisted of adding 70 % v/v HNO_3 and 30 % v/v H_2O_2 to the dried samples for an overnight pre-digestion (~12 h). The pre-digested samples were submitted to a microwave oven digestion. After cooling down, 37 % v/v HCl was added, samples were submitted once more to a microwave oven digestion, finally obtaining a clear solution indicative of a completely digested sample (the digestion program used is in Table S4).[1]

Table S4 - Digestion program used in the microwave oven for plant tissues.

	**Temperature (ºC)**	**Pressure (psi)**	**Ramp (ºC /min)**	**Time (min)**	**Power (watts)**
**1**	175	50	5	10	90
**2**	195	50	5	15	90
**3**	50	0	5	10	90
**4**	50	0	1	10	0
**5**	50	0	1	1	0

2.6. Zn distribution and speciation on pepper fresh tissues using Micro X-ray Fluorescence (µ-XRF) and Micro X-ray Absorption Near-Edge Structure (µ-XANES)

Roots and stems were embedded in OCT (optimal cutting temperature) resin and flash frozen in liquid nitrogen. Samples were cross-sectioned (20 µm thick) using a Leica cryo-

microtome (LN22), placed between two layers of Ultralene film and mounted on an in-house Cu sample-holder immediately after sectioning.

For XANES, an amount of 3 µL of each reference solution (1 µL for NPs) were pipetted between two layers of Ultralene film and mounted on the Cu sample-holder for analysis under cryogenic conditions.

Table S5 – Reference compounds used for Zn µ-XANES

Reference compound name	Functional group	References for synthesis method
**ZnO NPs**	Zn-O	Dybowska et al.[2]
**ZnO_Ph NPs**	Zn-o-Zn-O-P	Rathnayake et al.[3] and Muthukumaran and Gopalakrishnan[4]
**Zn-Phytate**	Zn-O-P-R	Asensio et al.[5]
**Zn-Cysteine**	Zn-S-R	Doan et al.[6]
**Zn-Histidine**	Zn-O-R	Provided by Dr. Geraldine Sarret (ISTerre, CNRS & University of Grenoble Alpes, France)
**Zn-Citrate**	Zn-O-R	Purchased from Sigma Aldrich ® (CAS 5990-32-9)

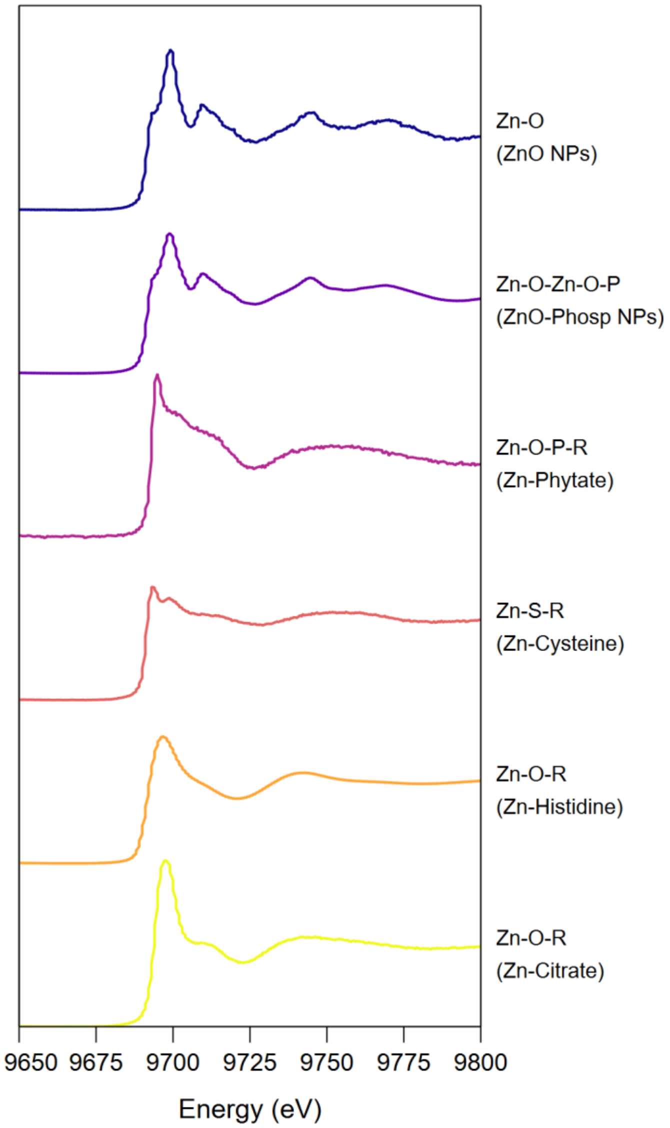

Figure S2 - Reference compounds used for Zn µ-XANES fitting and the simplified bonding environment used for the linear combination fittings. All reference compounds were analyzed at the ESRF ID21.

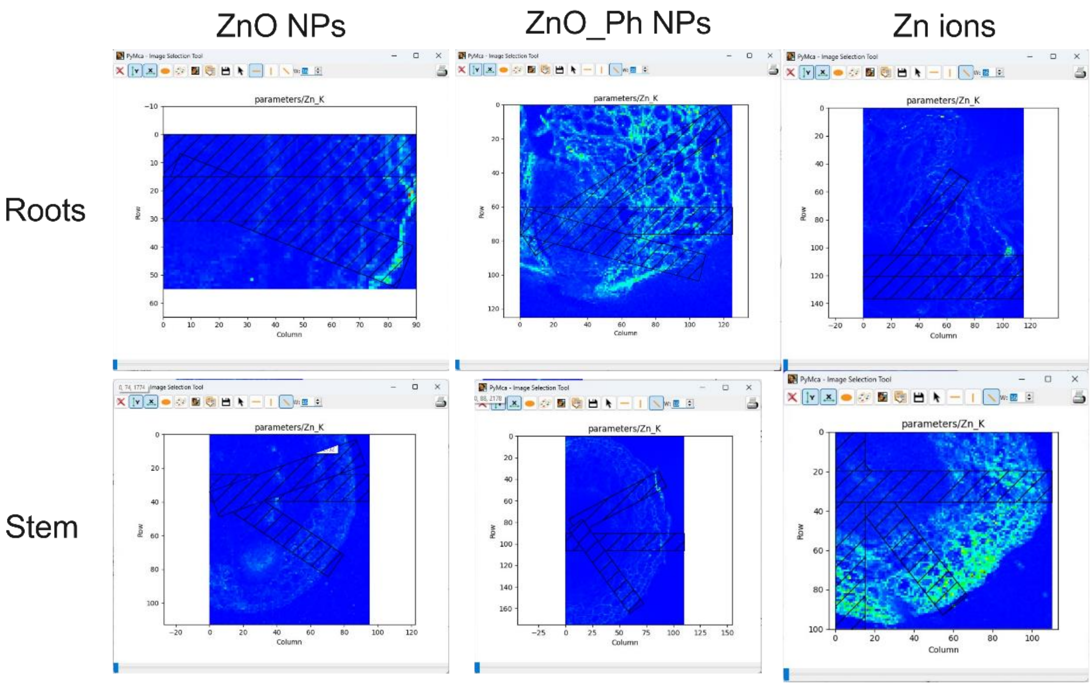

Figure S3 – Profiles selection for averaging the Zn intensity in roots and stems of plants exposed to ZnO NPs, ZnO_Ph NPs and Zn ions.

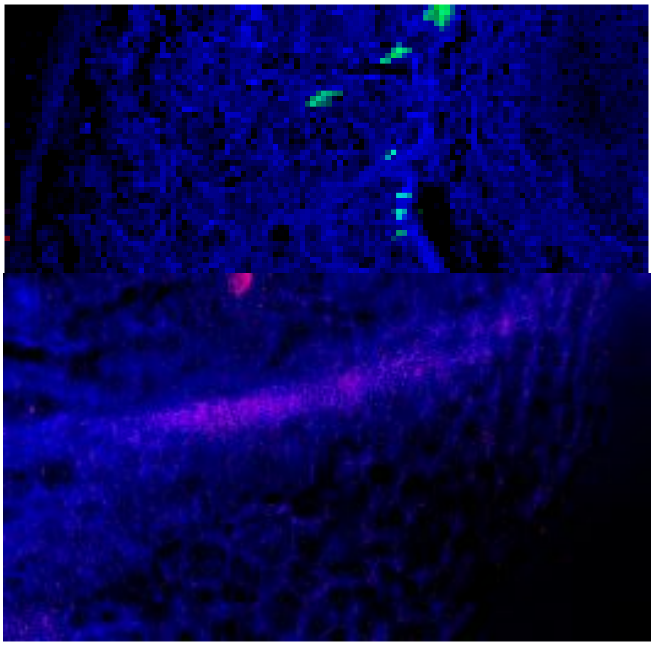

Figure S4 - µXRF elemental distribution map of the root (top) and stem (bottom) for the DIW control plant.

3. Results and discussion

3.1. Nanoparticle characterization and dissolution

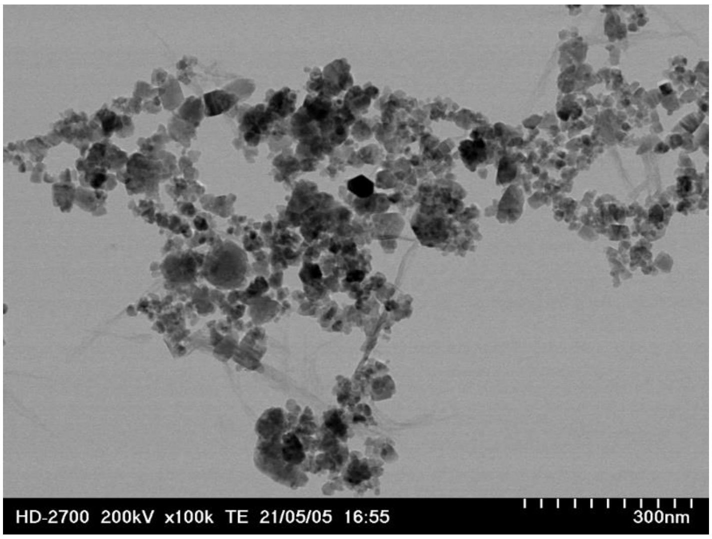

Figure S5 – TEM analysis of ZnO NP: Micrograph at 100Kx magnification.

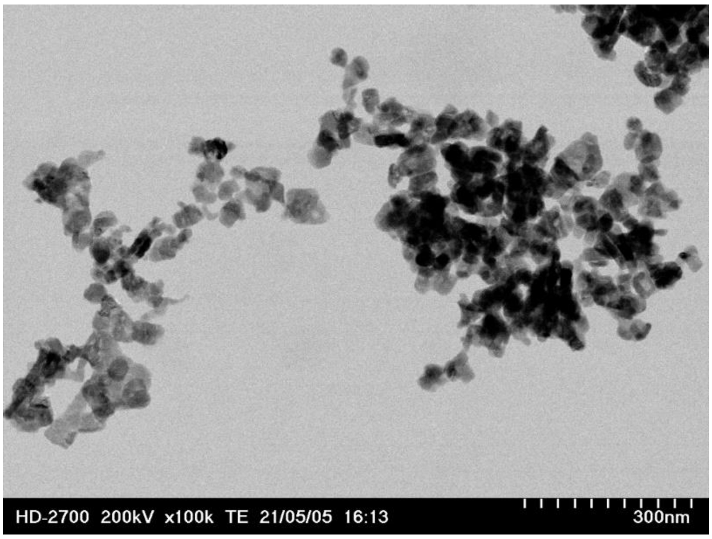

Figure S6– TEM analysis of ZnO_Ph NPs: Micrograph at 100Kx magnification.

Table S6 – ZnO NPs and ZnO_Ph NPs properties in MQ water

	TEM average nominal size (nm) [a, b]	Zeta potential (mV) [c]	Hydrodynamic diameter (nm) [b, c]	Zn (%w/w) [c]	pH of the medium
ZnO NPs	26 ± 8 [d]	14.6 ± 0.4 [d]	357 ± 126 [d]	89.9 ± 6.7 [d]	6.8 ± 0.2 [d]
ZnO_Ph NPs (2.0 ± 0.1% P) [c, d]	48 ± 12 [d]	-18.1 ± 0.6 [d]	317 ± 87 [d]	83.6 ± 1.1 [d]	6.8 ± 0.2 [d]

[a] Based on TEM images of at least 150 particles. [b] Intensity-weighted Z-average. [c] The results are presented as mean ± standard deviation (N=10 for zeta potential; N=3 for Zn and P%). [d] Values reported in Rodrigues et al.[1]. N/A – Not applicable.

3.2. ^{68}Zn root uptake and *in planta* translocation

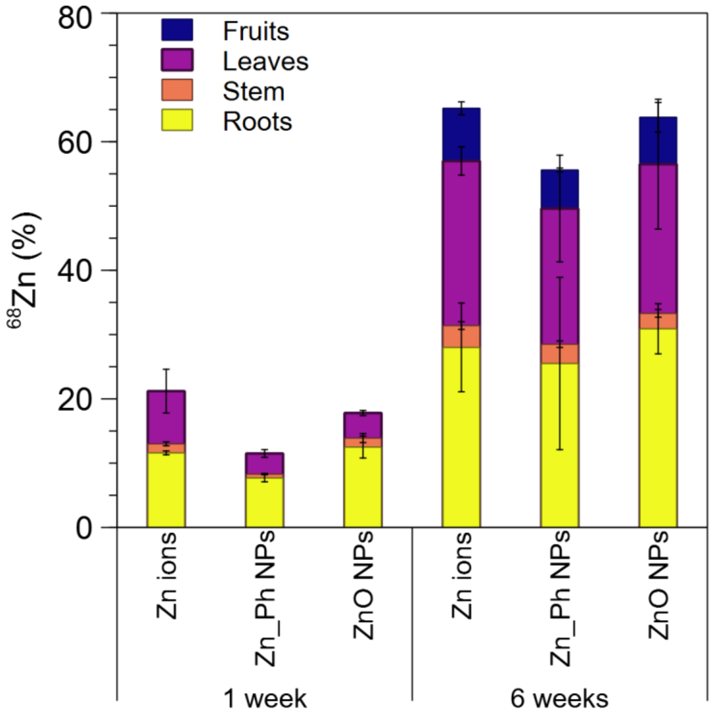

Figure S7- Translocation of ^{68}Zn (in % relative to the dose initially applied) to the roots, stem, leaves, and fruits of pepper plants, 1 week and 6 weeks after exposure to ZnO NPs, ZnO_Ph NPs and Zn ions. Three replicates per treatment were used to calculate the means and standard deviations (presented as error bars).

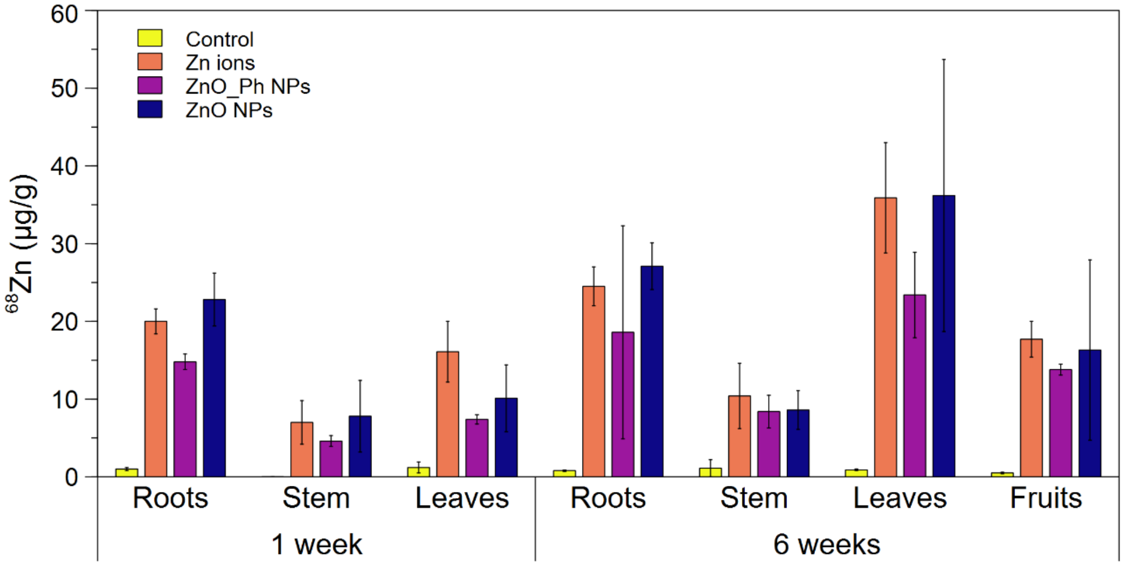

Figure S8 – Concentration of ^{68}Zn (in µg) in roots, stem, leaves and fruits of pepper plants, 1 week and 6 weeks after exposure to ZnO NPs, ZnO_Ph NPs and Zn ions. The control represented here comes from non-exposed pepper plants. Three replicates per treatment were used to calculate the means and standard deviations (presented as error bars).

3.3. Zn cellular distribution

Stats | Mean Ratio | Median Ratio | Pearson Correlation

	parameters/P_K	parameters/S_K	parameters/Cl_K	parameters/K_K	parameters/Ca_K	parameters/Ti_K	parameters/Mn_K	parameters/Fe_K	parameters/Ni_K	parameters/Cu_K	parameters/Zn_K
parameters/P_K	1.000	0.404	0.221	0.281	0.083	0.081	0.128	0.084	-0.016	0.121	0.143
parameters/S_K	0.404	1.000	0.425	0.550	0.126	0.114	0.298	0.165	-0.060	0.363	0.333
parameters/Cl_K	0.221	0.425	1.000	0.818	0.123	0.108	0.258	-0.040	-0.065	0.089	0.242
parameters/K_K	0.281	0.550	0.818	1.000	0.058	0.100	0.330	-0.003	-0.087	0.121	0.391
parameters/Ca_K	0.083	0.126	0.123	0.058	1.000	0.223	0.102	0.006	0.209	-0.004	0.031
parameters/Ti_K	0.081	0.114	0.108	0.100	0.223	1.000	0.100	0.090	0.042	0.080	0.087
parameters/Mn_K	0.128	0.298	0.258	0.330	0.102	0.100	1.000	0.208	-0.027	0.328	0.393
parameters/Fe_K	0.084	0.165	-0.040	-0.003	0.006	0.090	0.208	1.000	-0.006	0.411	0.277
parameters/Ni_K	-0.016	-0.060	-0.065	-0.087	0.209	0.042	-0.027	-0.006	1.000	-0.041	-0.017
parameters/Cu_K	0.121	0.363	0.089	0.121	-0.004	0.080	0.328	0.411	-0.041	1.000	0.350
parameters/Zn_K	0.143	0.333	0.242	0.391	0.031	0.087	0.393	0.277	-0.017	0.350	1.000

Figure S9 – Zn association to other elements in the root of the Zn ions treatment.

Stats | Mean Ratio | Median Ratio | Pearson Correlation

	parameters/P_K	parameters/S_K	parameters/Cl_K	parameters/K_K	parameters/Ca_K	parameters/Ti_K	parameters/Mn_K	parameters/Fe_K	parameters/Ni_K	parameters/Cu_K	parameters/Zn_K
parameters/P_K	1.000	0.363	0.242	0.323	0.002	0.007	0.018	0.007	-0.030	0.059	0.105
parameters/S_K	0.363	1.000	0.569	0.740	0.111	0.019	0.180	0.025	0.017	0.096	0.287
parameters/Cl_K	0.242	0.569	1.000	0.764	0.169	0.069	0.177	0.010	0.021	0.081	0.209
parameters/K_K	0.323	0.740	0.764	1.000	0.126	0.025	0.213	0.012	0.010	0.057	0.272
parameters/Ca_K	0.002	0.111	0.169	0.126	1.000	0.076	0.061	0.003	0.188	-0.042	0.026
parameters/Ti_K	0.007	0.019	0.069	0.025	0.076	1.000	0.066	0.777	0.034	0.079	0.110
parameters/Mn_K	0.018	0.180	0.177	0.213	0.061	0.066	1.000	0.107	0.061	0.076	0.259
parameters/Fe_K	0.007	0.025	0.010	0.012	0.003	0.777	0.107	1.000	0.030	0.190	0.327
parameters/Ni_K	-0.030	0.017	0.021	0.010	0.188	0.034	0.061	0.030	1.000	-0.028	0.050
parameters/Cu_K	0.059	0.096	0.081	0.057	-0.042	0.079	0.076	0.190	-0.028	1.000	0.287
parameters/Zn_K	0.105	0.287	0.209	0.272	0.026	0.110	0.259	0.327	0.050	0.287	1.000

Figure S10 – Zn association to other elements in the root of the ZnO NPs treatment.

Stats | Mean Ratio | Median Ratio | Pearson Correlation

	parameters/P_K	parameters/S_K	parameters/Cl_K	parameters/K_K	parameters/Ca_K	parameters/Ti_K	parameters/Mn_K	parameters/Fe_K	parameters/Ni_K	parameters/Cu_K	parameters/Zn_K
parameters/P_K	1.000	0.417	0.307	0.297	0.297	0.088	0.079	0.092	0.066	0.088	0.333
parameters/S_K	0.417	1.000	0.603	0.668	0.202	0.077	0.137	0.081	0.040	0.139	0.632
parameters/Cl_K	0.307	0.603	1.000	0.866	0.114	0.053	0.133	0.037	0.011	0.084	0.704
parameters/K_K	0.297	0.668	0.866	1.000	0.068	0.100	0.168	0.097	-0.006	0.112	0.827
parameters/Ca_K	0.297	0.202	0.114	0.068	1.000	0.121	0.078	0.025	0.263	0.029	0.168
parameters/Ti_K	0.088	0.077	0.053	0.100	0.121	1.000	0.102	0.512	0.040	0.031	0.084
parameters/Mn_K	0.079	0.137	0.133	0.168	0.078	0.102	1.000	0.144	0.091	0.057	0.175
parameters/Fe_K	0.092	0.081	0.037	0.097	0.025	0.512	0.144	1.000	0.030	0.055	0.082
parameters/Ni_K	0.066	0.040	0.011	-0.006	0.263	0.040	0.091	0.030	1.000	0.052	0.008
parameters/Cu_K	0.088	0.139	0.084	0.112	0.029	0.031	0.057	0.055	0.052	1.000	0.152
parameters/Zn_K	0.333	0.632	0.704	0.827	0.168	0.084	0.175	0.082	0.008	0.152	1.000

Figure S11 – Zn association to other elements in the root of the ZnO_Ph NPs treatment.

Stats | Mean Ratio | Median Ratio | Pearson Correlation

	parameters/P_K	parameters/S_K	parameters/Cl_K	parameters/K_K	parameters/Ca_K	parameters/Ti_K	parameters/Mn_K	parameters/Fe_K	parameters/Ni_K	parameters/Cu_K	parameters/Zn_K
parameters/P_K	1.000	0.458	0.333	0.374	-0.010	0.065	0.195	0.055	0.006	0.093	0.367
parameters/S_K	0.458	1.000	0.571	0.593	0.139	0.199	0.345	0.024	0.010	0.090	0.699
parameters/Cl_K	0.333	0.571	1.000	0.855	0.259	0.251	0.365	0.044	0.085	-0.013	0.748
parameters/K_K	0.374	0.593	0.855	1.000	0.168	0.232	0.422	0.042	0.012	0.070	0.837
parameters/Ca_K	-0.010	0.139	0.259	0.168	1.000	0.410	0.119	0.043	0.248	-0.008	0.056
parameters/Ti_K	0.065	0.199	0.251	0.232	0.410	1.000	0.223	0.135	0.140	0.020	0.185
parameters/Mn_K	0.195	0.345	0.365	0.422	0.119	0.223	1.000	0.768	0.108	0.205	0.425
parameters/Fe_K	0.055	0.024	0.044	0.042	0.043	0.135	0.768	1.000	0.216	0.125	0.036
parameters/Ni_K	0.006	0.010	0.085	0.012	0.248	0.140	0.108	0.216	1.000	-0.442	0.066
parameters/Cu_K	0.093	0.090	-0.013	0.070	-0.008	0.020	0.205	0.125	-0.442	1.000	-0.066
parameters/Zn_K	0.367	0.699	0.748	0.837	0.056	0.185	0.425	0.036	0.066	-0.066	1.000

Figure S12 – Zn association to other elements in the stem of the Zn ions treatment.

Stats | Mean Ratio | Median Ratio | Pearson Correlation

	parameters/P_K	parameters/S_K	parameters/Cl_K	parameters/K_K	parameters/Ca_K	parameters/Ti_K	parameters/Mn_K	parameters/Fe_K	parameters/Ni_K	parameters/Cu_K	parameters/Zn_K
parameters/P_K	1.000	0.653	0.219	0.318	-0.009	0.006	0.164	0.272	-0.095	0.082	0.332
parameters/S_K	0.653	1.000	0.462	0.609	0.167	0.066	0.483	0.264	-0.127	0.069	0.539
parameters/Cl_K	0.219	0.462	1.000	0.680	0.115	0.044	0.451	0.061	-0.084	-0.004	0.448
parameters/K_K	0.318	0.609	0.680	1.000	0.221	0.075	0.610	0.172	-0.153	0.011	0.592
parameters/Ca_K	-0.009	0.167	0.115	0.221	1.000	0.067	0.220	0.057	0.006	-0.010	0.128
parameters/Ti_K	0.006	0.066	0.044	0.075	0.067	1.000	0.080	0.021	0.007	-0.006	0.054
parameters/Mn_K	0.164	0.483	0.451	0.610	0.220	0.080	1.000	0.142	-0.092	0.010	0.508
parameters/Fe_K	0.272	0.264	0.061	0.172	0.057	0.021	0.142	1.000	-0.009	0.041	0.205
parameters/Ni_K	-0.095	-0.127	-0.084	-0.153	0.006	0.007	-0.092	-0.009	1.000	-0.050	-0.094
parameters/Cu_K	0.082	0.069	-0.004	0.011	-0.010	-0.006	0.010	0.041	-0.050	1.000	-0.012
parameters/Zn_K	0.332	0.539	0.448	0.592	0.128	0.054	0.508	0.205	-0.094	-0.012	1.000

Figure S13– Zn association to other elements in the stem of the ZnO NPs treatment.

Stats | Mean Ratio | Median Ratio | Pearson Correlation

	parameters/P_K	parameters/S_K	parameters/Cl_K	parameters/K_K	parameters/Ca_K	parameters/Ti_K	parameters/Mn_K	parameters/Fe_K	parameters/Ni_K	parameters/Cu_K	parameters/Zn_K
parameters/P_K	1.000	0.367	0.312	0.322	-0.000	0.022	0.068	0.078	-0.027	0.111	0.221
parameters/S_K	0.367	1.000	0.624	0.529	0.104	0.068	0.200	0.121	0.011	0.224	0.549
parameters/Cl_K	0.312	0.624	1.000	0.807	0.102	0.071	0.208	0.083	-0.006	0.141	0.543
parameters/K_K	0.322	0.529	0.807	1.000	0.063	0.067	0.189	0.064	-0.050	0.149	0.532
parameters/Ca_K	-0.000	0.104	0.102	0.063	1.000	0.201	0.011	0.018	0.401	-0.023	0.009
parameters/Ti_K	0.022	0.068	0.071	0.067	0.201	1.000	0.025	0.033	0.086	0.014	0.049
parameters/Mn_K	0.068	0.200	0.208	0.189	0.011	0.025	1.000	0.182	0.029	0.274	0.584
parameters/Fe_K	0.078	0.121	0.083	0.064	0.018	0.033	0.182	1.000	0.051	0.311	0.242
parameters/Ni_K	-0.027	0.011	-0.006	-0.050	0.401	0.086	0.029	0.051	1.000	0.014	0.002
parameters/Cu_K	0.111	0.224	0.141	0.149	-0.023	0.014	0.274	0.311	0.014	1.000	0.454
parameters/Zn_K	0.221	0.549	0.543	0.532	0.009	0.049	0.584	0.242	0.002	0.454	1.000

Figure S14 – Zn association to other elements in the stem of the ZnO_Ph NPs treatment.

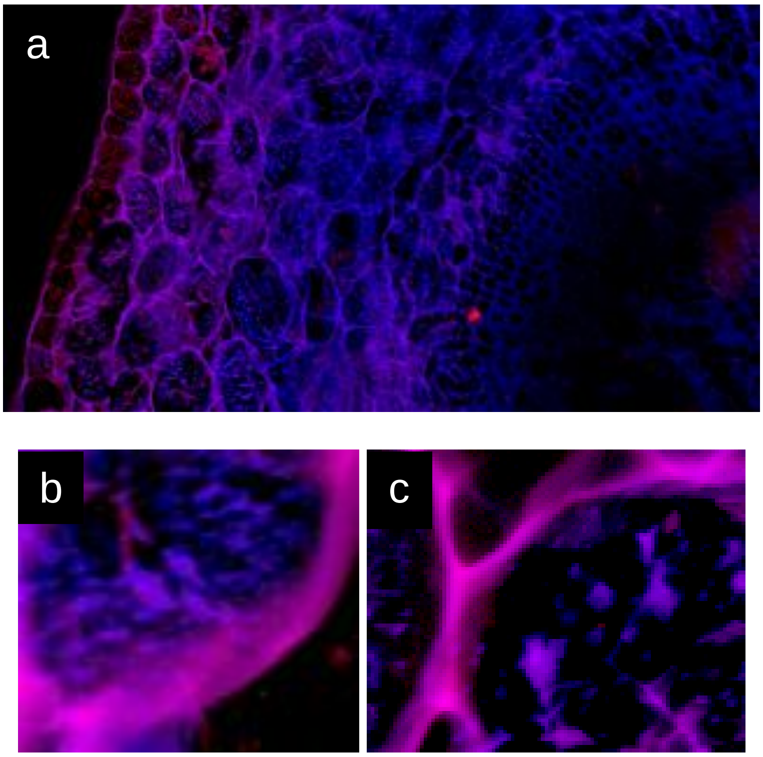

Figure S15 - Elemental µ-XRF map on (a) the stem of pepper plants exposed to ZnO_Ph NPs: one week after exposure. Picture (b) and (c) are close ups of epidermis cells of the same stem epidermis. The Kα fluorescence of Zn is represented in red and K in blue.

3.4. Zn *in planta* biotransformation and mobility following root uptake

Table S7 – Linear Combination Fitting of the µ-XANES spectra done in the different points of interest (POIs) on each cell tissue from the exposed roots and stem, 1 week after exposure. Lines in blue indicate the chosen combination fitting between two per cell type.

	Organ	Cell Type	Nº of POIs	Nº of components	ZnO NPs	ZnO_Ph NPs	Zn-Citrate	Zn-Histidine	Zn-Cysteine	Zn-Phytate	Total	Rf	$\chi2$	Reduced $\chi2$
**ZnOPh NPs**	Exposed root	Epidermis	26	3	-	-	16%	35%	-	50%	100%	8.84E-04	0.212	8.26E-04
**ZnOPh NPs**	Exposed root	Epidermis	26	2	-	-	-	56%	-	45%	101%	**1.10E-03**	**0.265**	1.03E-03
**ZnO NPs**	Exposed root	Epidermis	60	3	-	-	28%	35%	-	38%	100%	**3.48E-04**	**0.085**	3.30E-04
**ZnO NPs**	Exposed root	Epidermis	60	2	-	-	19%	83%	-	-	102%	1.90E-03	0.457	1.80E-03
**Zn ions**	Exposed root	Epidermis	19	2	-	-	98%	-	-	4%	102%	**7.89E-04**	**0.207**	7.71E-04
**Zn ions**	Exposed root	Epidermis	19	2	-	-	100%	2%	-	-	102%	8.37E-04	0.219	8.18E-04
**ZnOPh NPs**	Exposed root	Cortex	58	3	-	-	32%	-	33%	36%	100%	5.38E-04	0.127	4.95E-04
**ZnOPh NPs**	Exposed root	Cortex	58	2	-	-	-	66%	-	34%	100%	**9.59E-04**	**0.223**	8.65E-04

**ZnO NPs**	Exposed root	Cortex	20	3	-	-	31%	-	36%	34%	101%	**1.07E-03**	**0.253**	9.85E-04
**ZnO NPs**	Exposed root	Cortex	20	2	-	-	-	66%	-	34%	100%	2.25E-03	0.533	2.06E-03
**Zn ions**	Exposed root	Cortex	23	3	-	-	29%	37%	-	34%	100%	**6.16E-04**	**0.146**	5.68E-04
**Zn ions**	Exposed root	Cortex	23	2	-	-	48%	-	-	51%	99%	1.17E-03	0.283	1.10E-03
**ZnOPh NPs**	Exposed root	Vasculature	33	3	-	-	28%	-	33%	40%	101%	8.92E-04	0.213	8.28E-04
**ZnOPh NPs**	Exposed root	Vasculature	33	2	-	-	-	62%	-	38%	100%	**1.52E-04**	**0.363**	1.41E-03
**Zn ions**	Exposed root	Vasculature	16	3	-	-	18%	50%	-	32%	100%	**1.36E-03**	**0.326**	1.27E-03
**Zn ions**	Exposed root	Vasculature	16	2	-	-	-	71%	-	29%	101%	1.65E-03	0.396	1.54E-03
**ZnOPh NPs**	Stem	Epidermis	21	3	-	-	62%	24%	-	14%	100%	4.84E-04	0.118	4.60E-04
**ZnOPh NPs**	Stem	Epidermis	21	2	-	-	59%	42%	-	-	101%	**7.06E-04**	**0.172**	6.67E-04
**ZnO NPs**	Stem	Epidermis	22	3	-	-	-	31%	46%	23%	100%	**6.82E-04**	**0.155**	6.02E-04
**ZnO NPs**	Stem	Epidermis	22	2	-	-	-	53%	49%	-	101%	1.33E-03	0.302	1.17E-03
**Zn ions**	Stem	Epidermis	19	3	-	-	-	65%	18%	22%	105%	**9.32E-04**	**0.220**	8.57E-04
**Zn ions**	Stem	Epidermis	19	2	-	-	-	74%	-	29%	103%	1.36E-03	0.321	1.25E-03
**ZnOPh NPs**	Stem	Cortex	18	3	-	-	51%	33%	-	16%	100%	4.08E-04	0.099	3.84E-04
**ZnOPh NPs**	Stem	Cortex	18	2	-	-	47%	54%	-	-	101%	**7.03E-04**	**0.170**	6.59E-04
**ZnO NPs**	Stem	Cortex	13	3	-	-	-	30%	39%	32%	100%	**1.27E-03**	**0.293**	1.14E-03
**ZnO NPs**	Stem	Cortex	13	2	-	-	-	-	49%	50%	100%	1.96E-03	0.453	1.76E-03
**Zn ions**	Stem	Cortex	14	3	-	-	-	60%	12%	29%	101%	**1.21E-03**	**0.289**	1.12E-03
**Zn ions**	Stem	Cortex	14	2	-	-	-	69%	-	31%	100%	1.44E-03	0.342	1.33E-03
**ZnOPh NPs**	Stem	Vasculature	9	3	-	-	-	46%	21%	34%	100%	**4.65E-04**	**0.109**	4.24E-04
**ZnOPh NPs**	Stem	Vasculature	9	2	-	-	-	62%	-	37%	99%	1.21E-03	0.284	1.10E-03
**ZnO NPs**	Stem	Vasculature	14	3	-	-	-	37%	35%	28%	100%	**9.70E-04**	**0.225**	8.75E-04
**ZnO NPs**	Stem	Vasculature	14	2	-	-	-	63%	38%	-	102%	1.86E-03	0.432	1.67E-03
**Zn ions**	Stem	Vasculature	2	3	-	-	-	44%	23%	34%	101%	**5.84E-03**	**1.395**	5.43E-03
**Zn ions**	Stem	Vasculature	2	2	-	-	-	61%	-	38%	100%	6.73E-03	1.608	6.23E-03

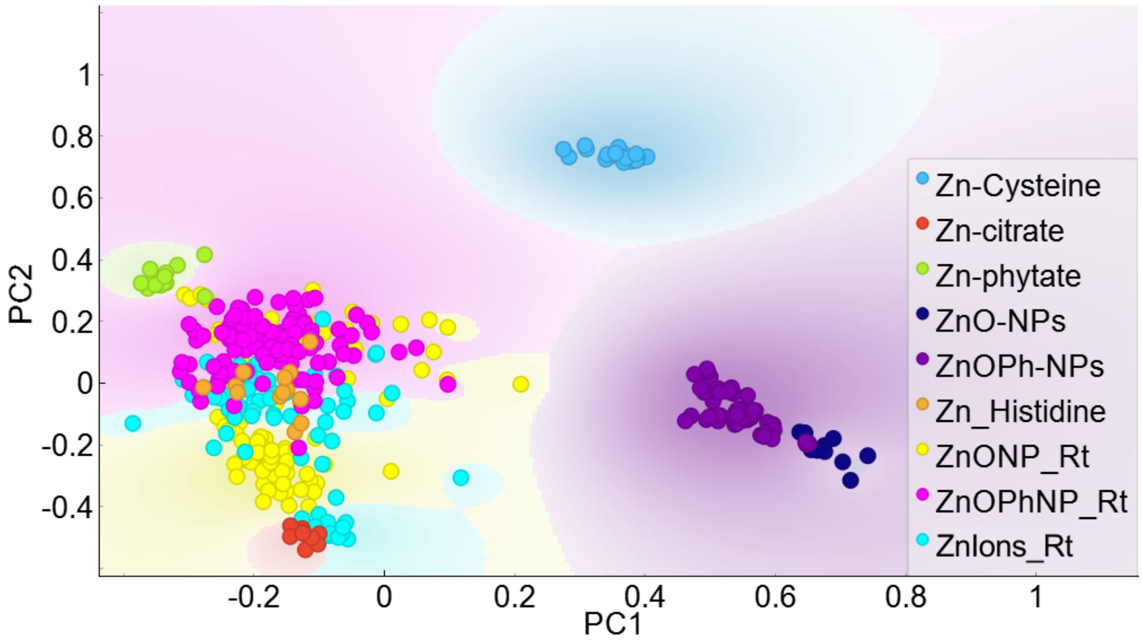

Figure S16 – PCA of the XANES done on all POIs in the exposed roots of pepper plants exposed to ZnO_Ph NPs (ZnOPhNP_Rt), ZnO NPs (ZnONP_Rt) or Zn ions (ZnIons_Rt) (1 week after exposure). The following references were used for comparison: Zn-Cysteine in blue (Zn-thiol), Zn-citrate in red (Zn-carboxyl), Zn-phytate in green (Zn-phosphate) and Zn-Histidine in orange (Zn-carboxyl). Eigenvalue for PC1: 0.52 and PC2: 0.38.

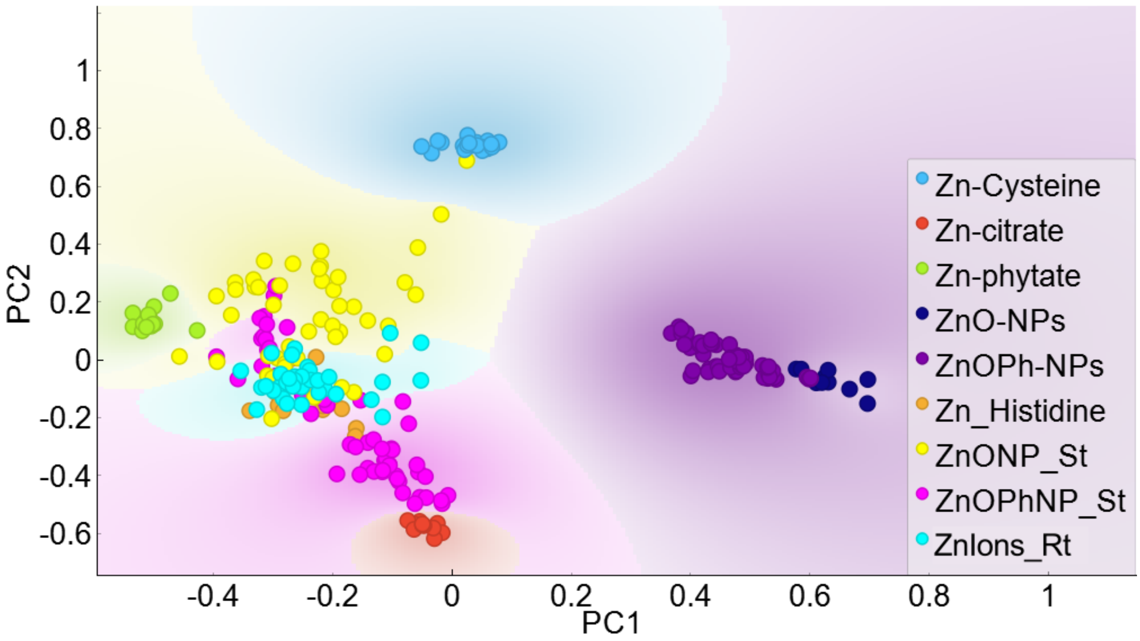

Figure S17 – PCA of the XANES done on all POIs in the stems of pepper plants exposed to ZnO_Ph NPs (ZnOPhNP_St), ZnO NPs (ZnONP_St) or Zn ions (ZnIons_St) (1 week after exposure). The following references were used for comparison: Zn-Cysteine in blue (Zn-thiol), Zn-citrate in red (Zn-carboxyl), Zn-phytate in green (Zn-phosphate) and Zn-Histidine in orange (Zn-carboxyl). Eigenvalue for PC1: 0.52 and PC2: 0.41.

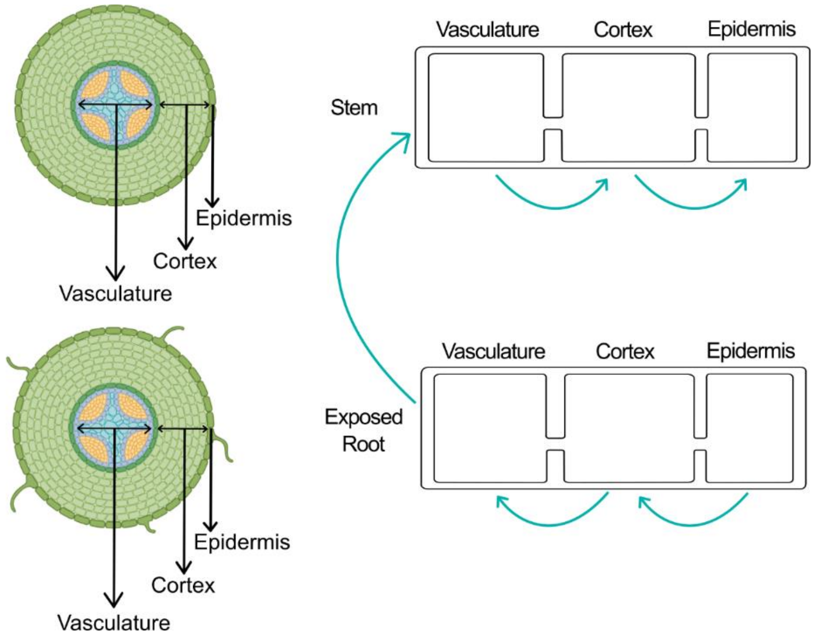

Figure S18 – Discussion flow scheme of the Zn speciation discussion (green arrows).

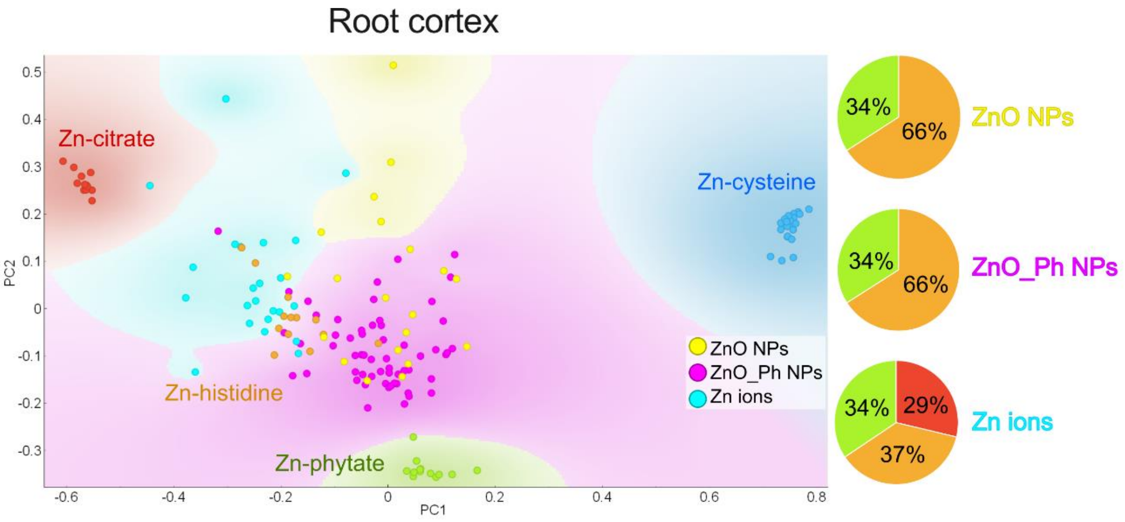

Figure S19 – PCA of the XANES done on selected POIs in the exposed root cortex of pepper plants exposed to ZnO_Ph NPs, ZnO NPs or Zn ions (1 week after exposure). The following references were used for comparison: Zn-Cysteine in blue (Zn-thiol), Zn-citrate in red (Zn-carboxyl), Zn-phytate in green (Zn-phosphate) and Zn-Histidine in orange (Zn-carboxyl). Eigenvalue for PC1: 0.69 and PC2: 0.18.

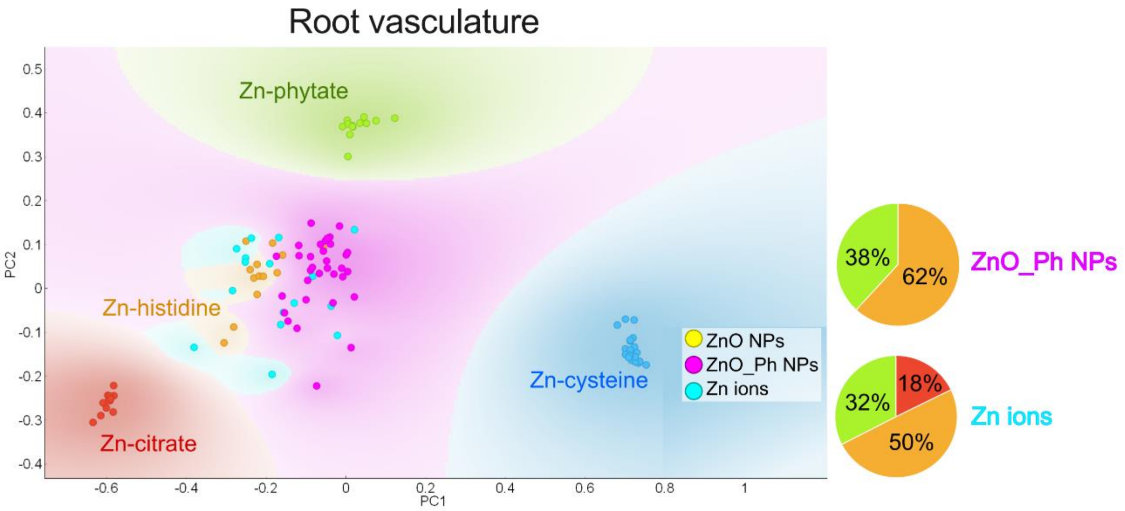

Figure S20- PCA of the XANES done on selected POIs in the root vasculature of pepper plants exposed to ZnO_Ph NPs, ZnO NPs or Zn ions (1 week after exposure). The following references were used for comparison: Zn-Cysteine in blue (Zn-thiol), Zn-citrate in red (Zn-carboxyl), Zn-phytate in green (Zn-phosphate) and Zn-Histidine in orange (Zn-carboxyl). Eigenvalue for PC1: 0.74 and PC2: 0.16.

3.5. Evaluation of Application strategies in Zn uptake and transport mechanisms

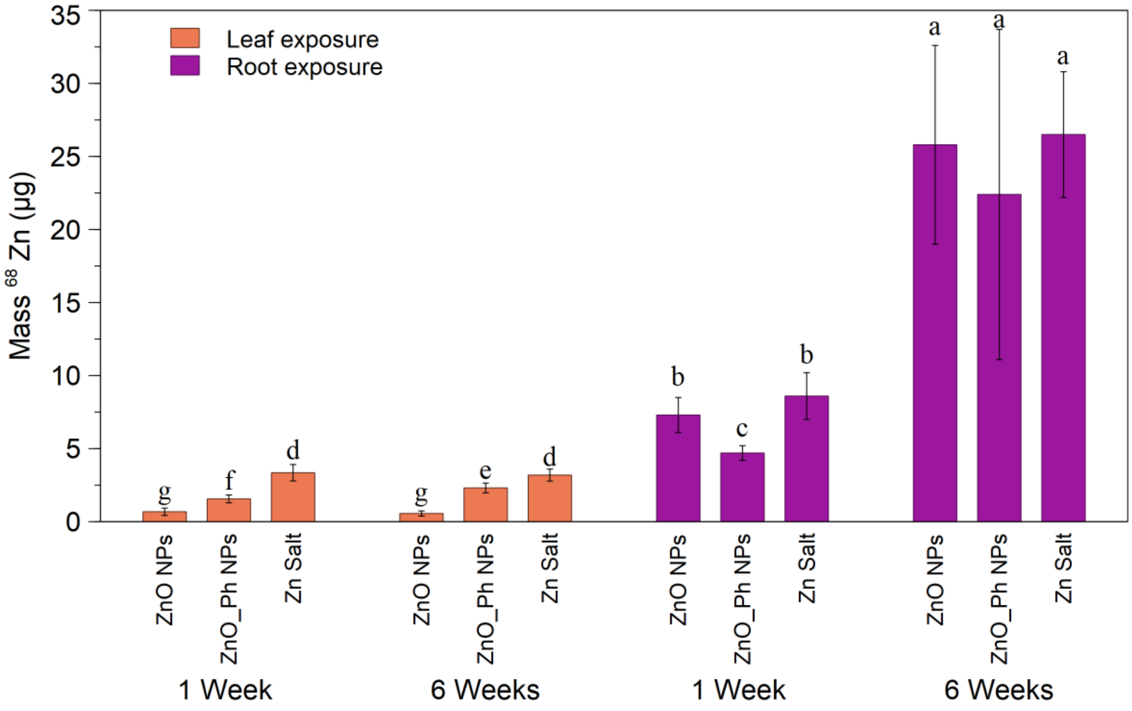

Figure S21 - ^{68}Zn mass taken up in the whole plant for both foliar and root exposure. Error bars represent the weighted standard deviation of the samples from three replicate plants. Statistically significant differences ($p < 0.05$) of the means of total ^{68}Zn masses for each treatment are indicated by different letters (on top of each bar chart).

References

(1) Rodrigues, S.; Avellan, A.; Bland, G. D.; Miranda, M. C. R.; Larue, C.; Wagner, M.; Moreno-Bayona, D. A.; Castillo-Michel, H.; Lowry, G. V; Rodrigues, S. M. Effect of a Zinc Phosphate Shell on the Uptake and Translocation of Foliarly Applied ZnO Nanoparticles in Pepper Plants (Capsicum Annuum). *Environ. Sci. Technol.* **2024**, *58* (7), 3213–3223. https://doi.org/10.1021/acs.est.3c08723.

(2) Dybowska, A.; Croteau, M.; Misra, S.; Berhanu, D.; Luoma, S.; Christian, P.; O'Brien, P.; Valsami-Jones, E. Synthesis of Isotopically Modified ZnO Nanoparticles and Their Potential as Nanotoxicity Tracers. *Environ. Pollut.* **2010**, *159*, 266–273. https://doi.org/10.1016/j.envpol.2010.08.032.

(3) Rathnayake, S.; Unrine, J. M.; Judy, J.; Miller, A.-F.; Rao, W.; Bertsch, P. M. Multitechnique Investigation of the PH Dependence of Phosphate Induced Transformations of ZnO Nanoparticles. *Environ. Sci. Technol.* **2014**, *48* (9), 4757–4764. https://doi.org/10.1021/es404544w.

(4) Muthukumaran, S.; Gopalakrishnan, R. Structural, FTIR and Photoluminescence Studies of Cu Doped ZnO Nanopowders by Co-Precipitation Method. *Opt. Mater. (Amst).* **2012**, *34* (11), 1946–1953. https://doi.org/https://doi.org/10.1016/j.optmat.2012.06.004.

(5) Asensio, G.; Hernández-Arriaga, A. M.; Martín-del-Campo, M.; Prieto, M. A.; Rojo, L.; Vázquez-Lasa, B. A Study on Sr/Zn Phytate Complexes: Structural Properties and Antimicrobial Synergistic Effects against Streptococcus Mutans. *Sci. Rep.* **2022**, *12* (1), 20177. https://doi.org/10.1038/s41598-022-24300-8.

(6) Doan, M. Y.; Worosz, M. A.; Cheek, G. T. Electrochemical Studies of Zinc/Cysteine Interactions. *ECS Meet. Abstr.* **2017**, *MA2017-01* (37), 1737–1737. https://doi.org/10.1149/ma2017-01/37/1737.
